# Multiple defects renovation and phase reconstruction of reduced-dimensional perovskites via in situ chlorination for efficient deep-blue (454 nm) light-emitting diodes

**DOI:** 10.1038/s41377-025-01768-3

**Published:** 2025-02-26

**Authors:** Mubing Yu, Tingxiao Qin, Gang Gao, Kelei Zu, Dongming Zhang, Nan Chen, Dengke Wang, Yong Hua, Hong Zhang, Yong-Biao Zhao, Jiaqi Zhu

**Affiliations:** 1 National Key Laboratory of Science and Technology on Advanced Composites in Special Environments, Harbin Institute of Technology, 150080 Harbin, China; 2 Zhengzhou Research Institute, Harbin Institute of Technology, 450046 Zhengzhou, China; 3 Beijing Academy of Quantum Information Sciences, 100193 Beijing, China; 4 Center for Optoelectronics Engineering Research, School of Physics and Astronomy, Yunnan University, 650500 Kunming, China; 5 Yunnan Key Laboratory for Micro/Nano Materials & Technology, School of Materials and Energy, Yunnan University, 650500 Kunming, China; 6 Key Laboratory of Micro-systems and Micro-structures Manufacturing Ministry of Education, Harbin Institute of Technology, 150080 Harbin, China

**Keywords:** Lasers, LEDs and light sources, Optical materials and structures

## Abstract

Deep-blue perovskite light-emitting diodes (PeLEDs) based on reduced-dimensional perovskites (RDPs) still face a few challenges including severe trap-assisted nonradiative recombination, sluggish exciton transfer, and undesirable bathochromic shift of the electroluminescence spectra, impeding the realization of high-performance PeLEDs. Herein, an in situ chlorination (isCl) post-treatment strategy was employed to regulate phase reconstruction and renovate multiple defects of RDPs, leading to superior carrier cooling of 0.88 ps, extraordinary exciton binding energy of 122.53 meV, and higher photoluminescence quantum yield of 60.9% for RDP films with deep-blue emission at 450 nm. The phase regulation is accomplished via fluorine-derived hydrogen bonds that suppress the formation of small-*n* phases. Multiple defects, including halide vacancies (shallow-state defects) and lead-chloride antisite defects (deep-state defects), are renovated via C=O coordination and hydroxy-group-derived hydrogen bonds. Consequently, deep-blue PeLEDs with a record maximum external quantum efficiency of 6.17% and stable electroluminescence at 454 nm were demonstrated, representing the best-performing deep-blue PeLEDs.

## Abstract (web summary)

In situ chlorination strategy was proposed to renovate multiple defects along with reconstruction of phases in RDPs for efficient and spectrally stable deep-blue PeLEDs with a record EQE of 6.17%.

## Graphical abstract

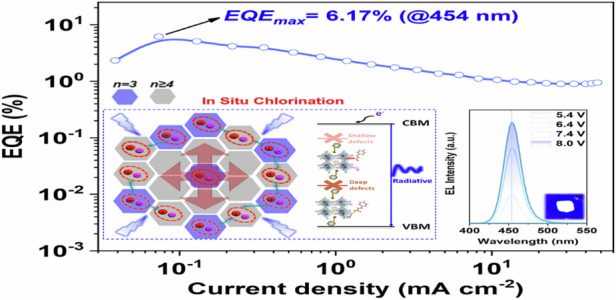

## Introduction

Metal halide perovskites exhibit significant potential for high-performance perovskite light-emitting diodes (PeLEDs) due to their high color purity, tunable bandgap, cost-effectiveness, and solution processability^1,2^. Numerous efforts on composition engineering, surface defect passivation as well as device optimization were devoted to improving the performance of PeLEDs, and the external quantum efficiencies (EQEs) of green, red, near-infrared, and even sky-blue PeLEDs are approaching or even higher than 20%^3–5^. In contrast, deep-blue emitters, especially for *λ* < 460 nm (compared with the commercial GaN counterparts), which are crucial for achieving the Rec. 2100 standard to realize ultrahigh-definition displays, still lag behind^6^. Among different strategies to achieve blue emitters, reduced-dimensional perovskites (RDPs) have attracted increasing attention for blue-PeLED applications. Due to the comprehensive effects of spatial confinement and dielectric confinement, reduced-dimensional perovskites (RDPs) show larger exciton binding energy and higher photoluminescence quantum yields (PLQYs)^7^.

There are a few factors hindering the development of deep-blue RDP emitters. One common issue is the heterogeneous phase distribution of various-*n* phases, which generally results in the tardy energy transfer from small-*n* phases to high-*n* phases and, thus, inefficient radiative recombination. Another issue is severe trap-assisted nonradiative recombination induced by halide vacancies (shallow-state defects), lead-lead clusters, and lead-halide antisite defects (deep-state defects) resulting from facile ion migration due to high chlorine concentration^8,9^. Therefore, considerable efforts have been directed towards controlling the crystallization kinetics, diminishing nonradiative recombination, and improving the stability of RDPs for blue emission^10–12^. Nevertheless, more simple and feasible strategies that could simultaneously realize multiple defects renovation both in the bulk and on the surface with shallow and deep state, along with precise phase distribution modification of RDPs for deep-blue emission, are yet to be proposed.

Herein, we report an in situ chlorination (isCl) post-treatment strategy by utilizing p-fluorocinnamoyl chloride (p-FCACl) dissolved in antisolvent to regulate the crystallization kinetics of RDPs with a composition of PEA_2_(Cs_*x*_EA_1-*x*_PbBr_y_Cl_3-*y*_)_2_PbBr_4_ (PEA = phenylethylamine, EA = ethylamine). Comprehensive characterization reveals that p-fluorocinnamic acid (p-FCA), the byproduct of isCl, interacts with halide vacancies and lead-chloride antisite defects to renovate multiple defects. Meanwhile, it allows the reconstruction of phase distribution by thoroughly suppressing the formation of small-*n* domains, leading to an ultrafast energy transfer. Moreover, the released chloride ions during isCl passivate halide vacancies in the bulk, inducing significantly blue-shifted emission ranging from 457 nm to 447 nm. Consequently, the optoelectronic properties and stability of RDPs are significantly improved, resulting in a record EQE of 6.17% for deep-blue emission at 454 nm.

## Results

### Device performance

**Fig. 1:**
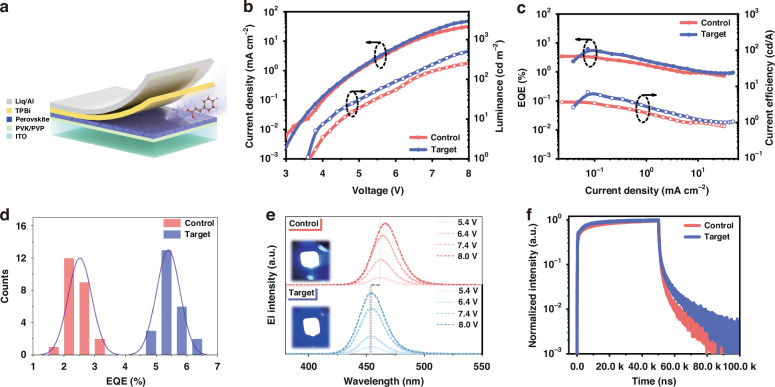
Device structure and performance of PeLEDs. **a** Schematic diagram of the PeLEDs structure. **b** Current density and luminance as a function of applied voltage of the PeLEDs. **c** EQE and CE as a function of the current density of the PeLEDs. **d** Statistical EQEs of the PeLEDs. **e** EL spectra of the PeLEDs. Inset shows the photograph of the corresponding PeLED operating at an applied voltage of 7.0 V. **f** TREL spectra of the PeLEDs

Deep-blue PeLEDs with a structure of ITO/PVK (30 nm)/PVP/RDPs (20 nm)/TPBi (35 nm)/Liq (2 nm)/Al (100 nm), as illustrated in Fig. 1a, were utilized to confirm the effect of isCl method. Mixed chlorine/bromine RDPs with a structure of PEA_2_(Cs_*x*_EA_1-*x*_PbBr_*y*_Cl_3-*y*_)_2_PbBr_4_ were utilized as the emitting layer prepared by typical one-step crystal-pinning method with antisolvent. RDPs treated without isCl and with isCl (p-FCACl, 3 mg mL^−1^) are served as the isCl-0 (control) and isCl-3 (target) samples, respectively. The cross-sectional SEM image of the device is presented in Fig. S1 (Supporting Information). Fig. S2a–d (Supporting Information) presents the UPS spectra of the isCl-0 and isCl-3 samples for determining the work function and the valence band maximum (VBM) of both samples. Furthermore, there are additional states between the VBM and the Fermi level, which are likely due to deep-state defects (Fig. S2e, Supporting Information). The weakening of these midgap states in the isCl-treated sample may be related to the renovation of the deep-state defects, which will be explained in detail later^13^. The bandgap of both RDP samples and the energy levels of each other layer in the PeLED are depicted in Fig. S3 (Supporting Information) and Fig. S4 (Supporting Information), respectively^14^. The *J–V–L* curves of the isCl-0 and isCl-3 PeLED are presented in Fig. 1b. It can be seen that the isCl-3 PeLED exhibits suppressed leakage current with a slightly smaller turn-on voltage (*V*_*on*_) and a maximum luminance of 510 cd m^−2^, which is higher than that of the isCl-0 device (254 cd m^−2^). This results in a maximum EQE of 6.17% and a maximum current efficiency (CE) of 7.02 cd A^−1^, corresponding to a 1.78-fold improvement of the isCl-0 device (Fig. 1c). This efficiency is the highest value for deep-blue PeLEDs based on RDPs with electroluminescence (EL) peak in the range of 445 to 458 nm (Fig. S5, Supporting Information). Furthermore, Fig. 1d presents the statistical EQEs of 24 PeLEDs for both isCl-0 and isCl-3 devices, which present an average EQE of 2.52% and 5.10%, respectively, indicating favorable reproducibility.

As depicted in Fig. 1e, it is worth mentioning that the isCl-0 PeLED exhibits an obviously bathochromic shift from 461 nm to 466 nm in the EL spectra as the bias ramps up to 8.0 V accompanied by a widened full width at half maximum (FWHM) of 26 nm. In contrast, the isCl-3 PeLED shows unchanged EL spectra at 454 nm with a narrowed FWHM of 24 nm, corresponding to a stable Commission International de l′Eclairage (CIE) coordinate of (0.149, 0.025) (Fig. S6a, b, Supporting Information). Such satisfactory enhancement in stability and blue-shift of the EL spectra results from the suppressed halide ion migration and released chloride ions enlarging the bandgap of RDPs. Besides, the operational stability of the unencapsulated PeLED was tested, and the decay curves are shown in Fig. S6c (Supporting Information), which reveals that the isCl-3 PeLED exhibits a prolonged half-lifetime (*T*_*50*_) of 24.9 min at a constant current density of 0.07 mA cm^−2^ (@ peak EQE position), which is about a 4-fold improvement over the isCl-0 PeLED (6.5 min). Time-resolved EL (TREL) measurements were conducted to qualitatively compare the charge injection and defects of the isCl-0 and isCl-3 PeLEDs with an impulse voltage of 6.0 V (Fig. 1f). It can be found that isCl-3 device demonstrated a faster response than isCl-0 device before the saturation stage, which benefits the slightly decreased *V*_*on*_ of the isCl-3 device^15,16^. Moreover, we fitted the curve consisting of fast and slow decay regions in the falling edge of the TREL spectra, which represents the depletion for radiative recombination and trap-assisted nonradiative recombination, respectively. As shown in Table S1 (Supporting Information), the increase in decay lifetime from 1.28 μs (isCl-0) to 2.96 μs (isCl-3) suggests enhanced radiative recombination and fewer defects to suppress photon quenching, which contributes to higher luminance and prolonged operation lifetime of the isCl-treated device^5^.

### Optoelectronic properties of reduced-dimensional perovskites

DFT computation (Table S2, Supporting Information) and ^13^C nuclear magnetic resonance (NMR) spectroscopy (Fig. S7, Supporting Information) suggest that p-FCACl enables to release of chloride ions and transform into p-FCA during isCl. In the following contents, we will show how the chloride ions and p-FCA affect the resulting RDPs.

**Fig. 2:**
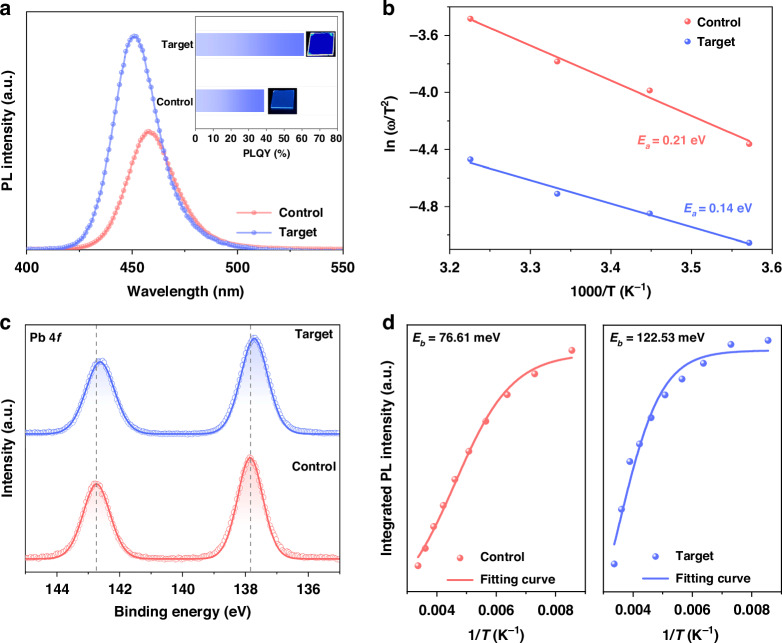
Optoelectronic properties of RDP films. **a** PL spectra of the RDP films. Inset shows the PLQYs and photographs under ultraviolet illumination of the RDP films. **b** Arrhenius plots of the characteristic frequencies to extract the defect activation energy (*E*_*a*_) for the RDP films. **c** Core-level XPS spectra of Pb 4 *f* of the RDP films. **d** Integration of the temperature-dependent PL intensity of the RDP films and fitting curves for *E*_*b*_

Comprehensive spectroscopic characterization techniques were used to understand the effects of isCl. By tuning the concentration of p-FCACl in the antisolvent, steady-state photoluminescence (PL) peak could be adjusted in the range of 457 nm to 447 nm (Fig. S8a, Supporting Information), indicating isCl indeed provides chloride ions towards RDPs^11,17^. The isCl-3 sample shows a record PLQY of 60.9%, which is much higher than that of 38.6% for the isCl-0 sample (Fig. 2a). Time-resolved PL (TRPL) measurements (Fig. S8b, Supporting Information) suggest a prolonged average carrier lifetime (${\tau }_{{avg}}$) of 10.94 ns for the isCl-3 sample, compared with that of 4.55 ns for the isCl-0 sample (Table S3, Supporting Information). Accordingly, the nonradiative recombination rate decreases from 2.11 × 10^8 ^s^−1^ (isCl-0) to 9.14 × 10^7 ^s^−1^ (isCl-3), indicating the nonradiative recombination is largely suppressed due to enhanced defect renovation by isCl treatment (Note 1, Supporting Information). However, there is a decrease in the PLQY and ${\tau }_{{avg}}$ of the sample treated with 4 mg mL^−1^ p-FCACl (Fig. S8c, Supporting Information), which is attributed to the slightly increased defects due to a higher Cl/Br ratio in the resulting RDPs^18,19^.

Temperature-dependent admittance spectra (AS) were used to analyze the energy distribution of defects, and the corresponding capacitance-frequency spectra are shown in Fig. S9 (Supporting Information)^20,21^. From the slope of ln (ω_0_ T^−2^) versus T^−1^ plots (Fig. 2b), the defect activation energy (*E*_*a*_) (Note 2, Supporting Information) decreases from 0.21 eV for the isCl-0 device to 0.14 eV for the isCl-3 device, indicating that p-FCACl could renovate deep-state defects^22–24^. Moreover, X-ray Photoelectron Spectroscopy (XPS) measurements (Fig. 2c) reveal a downward shift in Pb 4*f* for the isCl-3 sample, consistent with previous literature^25–27^. Meanwhile, Fourier transform infrared spectra (FTIR) (Fig. S10, Supporting Information) show a noticeable shift in the C=O stretching vibration peak (υ_C=O_) from 1759 to 1732 cm^−1^ for the isCl-3 sample, indicating there are interactions of C=O groups with uncoordinated lead^28,29^.

Temperature-dependent PL measurements were utilized to investigate the exciton binding energy (*E*_*b*_), as shown in Fig. S11 (Supporting Information)^30^. In all cases, we observe a similar phenomenon: increased PL intensity, red-shifted peak positions, and narrowed FWHM with decreasing temperature. This suggests the suppression of exciton-phonon coupling and thermal-assisted nonradiative recombination^31^. The fitted *E*_*b*_ using the Arrhenius equation (Note 3, Supporting Information) indicates there is a significant increase from 76.61 meV for the isCl-0 sample to 122.53 meV for the isCl-3 sample (Fig. 2d)^32^, suggesting efficient elimination of single-molecule recombination and increased barriers for exciton dissociation into free carriers^33^. This contributes to pronounced exciton radiative recombination and agrees well with the result of prolonged decay lifetime in the TREL spectra and higher PLQY for the isCl-treated sample.

Kelvin probe force microscopy (KPFM) measurements (Fig. S12a, b, Supporting Information) show a significant increase in the surface potential of the isCl-3 sample, indicating improved renovation of surface defects^34^. Indeed, space-charge-limited current (SCLC) analysis (Fig. S12c, Supporting Information) reveals decreased trap-filled limiting voltages (*V*_TFL_) of the isCl-3 device from 0.34 V to 0.24 V, and the corresponding trap density decreases from 1.81 × 10^17 ^cm^−3^ (the isCl-0 device) to 1.27 × 10^17 ^cm^−3^ (the isCl-3 device)^35^.

The moisture stability of the isCl-0 and isCl-3 samples was investigated in the ambient atmosphere with relative humidity (RH) of 68–70% (Fig. S13a, Supporting Information). The isCl-0 sample lost about 70% of initial PLQY after 6 h accompanied with a red-shifted peak of 5 nm, instead, the isCl-3 sample sustained half of initial PLQY with negligible peak shift. We found the isCl-3 sample showed a larger water contact angle (CA) (Fig. S13b, c, Supporting Information) of 37° than that of the isCl-0 sample (25°), indicating improved hydrophobicity^36–38^.

### Mechanism study of multiple defects renovation

Above photophysical findings indicate isCl helps to renovate both shallow- and deep-state defects, however, the underlying molecular mechanism is not clear. In this section, we use combined DFT calculation and chemical analysis to study how isCl might change the local interactions.

**Fig. 3:**
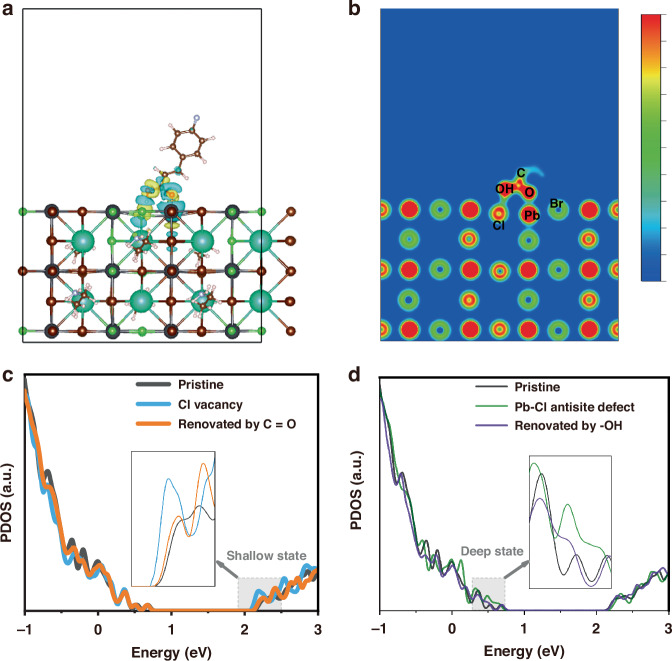
Theoretical calculations of multiple defects renovation. **a** Calculated perovskite combined with p-FCA, along with the corresponding differential charge density distribution. **b** Electron localization function of p-FCA treated perovskite. **c** DOS curves of the pristine perovskite, perovskite containing a chloride vacancy and renovated by C=O group. **d** DOS curves of the pristine perovskite, perovskite containing a lead-chloride defect and renovated by hydroxy group

As reported by previous literature, halide ion migration may create halide vacancies and lead-halide antisite defects^17,39^. The key to suppressing these defects is to increase the halide bonding strength of the perovskite frameworks. Thus, we first would like to discuss how isCl might enhance this effect. We utilized DFT calculation to get the optimized structure of the pristine perovskites (Fig. S14a, Supporting Information). The slab models of perovskites combined with p-FCA and the corresponding differential charge density distribution are shown in Fig. 3a. It can be seen that the uncoordinated lead is surrounded by a distinct cyan cloud (referring to electron depletion) in the model attached by C=O in p-FCA, illustrating strong interaction. Meanwhile, the chloride ion is surrounded by a distinct yellow cloud (referring to electron accumulation), suggesting the hydrogen bond between hydroxy group in p-FCA and halide ions. Notably, we compare two systems in terms of perovskites combined with and without such hydrogen bond (Fig. S14b, Supporting Information), and find that there is an increase in *E*_*b*_ from 1.06 eV to 1.57 eV, indicating stronger anchor due to the hydrogen bond^40,41^. Besides, electron localization function (ELF) calculations were performed to offer more detailed information at the atom scale (Fig. 3b)^42^. Such distorted electron clouds between the lead ion and C=O group, along with distorted electron clouds between the chloride ion and hydroxy group, further verify these interactions.

To justify the above theoretical analysis, interactions of pure p-FCA with each precursor component (CsCl, PbBr_2_, PEABr, and EACl) were studied by ^13^C NMR spectroscopy by dissolving individual components in deuterated DMSO. As presented in Fig. S15 (Supporting Information), the resonance signals of p-FCA mixed with CsCl, PEABr, and EACl exhibited an up-field shift compared with pure p-FCA, suggesting there might exist a de-shielding effect that reduces the density of the electron cloud around the carbon nucleus due to the formation of the hydroxyl-group-derived hydrogen bonds^43^. In contrast, the resonance signals of p-FCA mixed with PbBr_2_ show a little down-field shift compared to pure p-FCA. Such different changes may be attributed to the combined effect of coordination (that reduces the electron density around the carbon atom) and the presence of such hydrogen bonds^17^. Moreover, the XPS spectra of the Br 3*d* and Cl 2*p* for the treated sample offer a tendency towards larger binding energy with a 0.10 eV and 0.09 eV shift (Fig. S16a, b, Supporting Information), respectively, further indicating comprehensive effects of such hydrogen bonds and coordination.

Furthermore, projected density of states (DOS) calculations were used to study how the above interactions might change the defect distributions. To simplify the analysis, four slab models with a chloride vacancy, a lead-chloride antisite defect, renovated by C=O group and renovated by the hydroxy group in p-FCA were constructed (Fig. S17, Supporting Information)^41^. As shown in Fig. 3c, compared with the pristine sample, a chloride vacancy could introduce localized shallow-state defects below the conduction band minimum (CBM). After renovating by C=O group in p-FCA, the DOS of these shallow-state defects were substantially diminished. Meanwhile, as shown in Fig. 3d, compared with the pristine sample, a lead-chloride antisite defect could introduce localized deep-state defects in the bandgap above the VBM, consistent with previous literature^42^. This kind of defect resulting from ion migration could be eliminated by hydroxy-group-derived hydrogen bonds^43^, and the resultant DOS of deep-state defects is indeed alleviated. Such full defect renovation attributed to the suppressed formation of halide vacancies and halide ion migration contributes to eliminating carrier capture in multiple defects, thus facilitating radiative recombination to enhance the luminance and prolong the lifetime of isCl-treated device.

### Phase reconstruction of reduced-dimensional perovskites

**Fig. 4:**
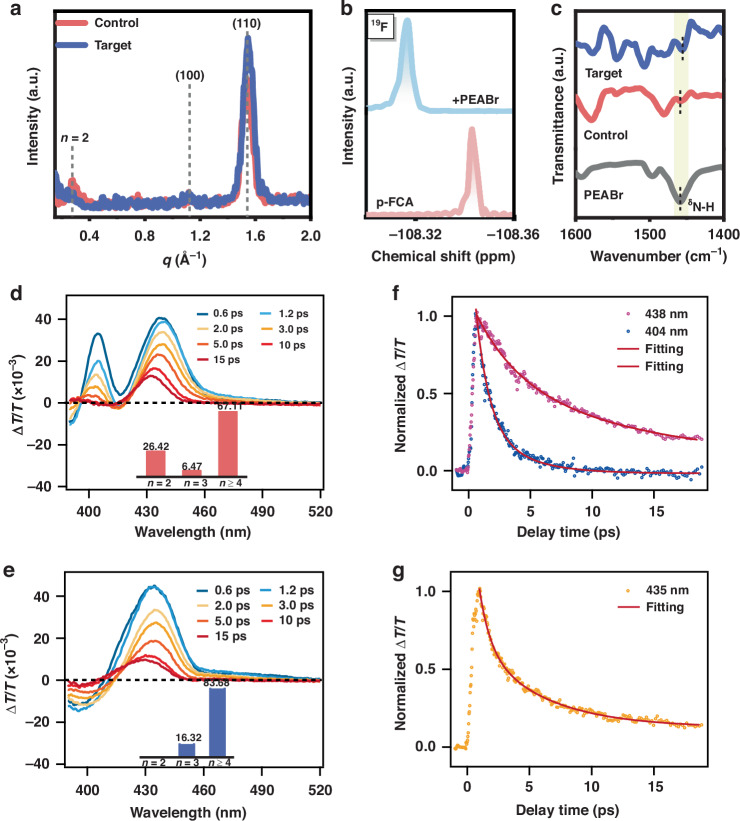
Phase reconstruction and energy transfer dynamics of RDP films. **a** Line integral curve of the GIWAXS patterns of the RDP films. **b** ^19^F NMR spectra of pure p-FCA and p-FCA mixed with PEABr. **c** FTIR spectra of pure PEABr, the control and target RDP film. TA spectra at various specific timescales of the **d** control and **e** target RDP film. The insert is the relative content of each phase in the RDP films. TA kinetics **f** at 404 nm and 438 nm of the control and **g** at 435 nm of the target RDP film

The isCl treatment was also found to have a profound effect on the phase distribution of the RDPs. As shown in Fig. S18a (Supporting Information), the isCl-0 sample shows the multiple exciton absorption peaks around 404 nm, corresponding to *n* = 2 domains. During isCl, this absorption peak is completely disappeared, and the absorption spectra resembles more that of quasi-3D perovskites, suggesting isCl regulates the crystallization kinetics^14^. Moreover, there is an obvious blue-shift in the absorption edge of isCl-treated sample, suggesting an enlarged bandgap resulting from chloride insertion towards perovskite frameworks, which agrees well with such blue-shift of the peak in the PL spectra. Grazing-incidence wide-angle X-ray scattering (GIWAXS) measurements were carried out to investigate the phase distribution and crystal orientation of RDPs. As illustrated in Fig. S18b, c (Supporting Information), the Debye-Scherrer scattering rings at *q* = 1.12 Å^−1^ and 1.54 Å^−1^ belonging to (100) and (110) crystal plane of 3D perovskites^44^, exhibit a slightly bathochromic shift in comparison with the isCl-0 sample (Fig. 4a), indicating a reduction in crystal lattice size as more chloride ions enter perovskite frameworks^12^. Moreover, the intensity of the diffraction associated with the (110) crystal plane becomes notably stronger, indicating there is a preferred orientation^45^. Additionally, a conspicuous reduction in the intensity of scattering rings at *q* = 0.27 Å^−1^, corresponding to the (010) crystal plane of *n* = 2 domains, could be observed, suggesting the efficient suppression of small-*n* phases. isCl also impacts the morphology of RDPs. Scanning electron microscopy (SEM) and atomic force microscopy (AFM) images (Fig. S19, Supporting Information) indicate a more compact structure, fewer pinholes, and reduced root-mean-square (RMS) roughness from 7.02 nm (isCl-0) to 3.85 nm (isCl-3).

To understand the phase regulation mechanism, ^19^F NMR spectroscopy of deuterated DMSO solutions of pure p-FCA with and without PEABr was studied (Fig. 4b). The signal presented a down-field-shifted after the addition of PEABr, indicating the hydrogen bonds between the fluorine atoms and the organic cations^46^. This interaction was further supported by FTIR and XPS results. In the FTIR spectra, the N-H bending vibration peak (δ_N-H_) of the isCl-3 sample exhibits a downshift from 1459 cm^−1^ to 1455 cm^−1^ compared with pure PEABr (Fig. 4c). The N 1 *s* core-level XPS spectra with and without isCl treatment (Fig. S16c, Supporting Information) shows a noticeable up-shift in binding energy. This interaction would prevent the adsorption of organic cations to the perovskite frameworks, suppressing the formation of small-*n* phases^47^.

Phase reconstruction was found to significantly alter the carrier dynamics, as revealed by transient absorption (TA) spectroscopy. Fig. S20a, b (Supporting Information) presents the TA color maps of the isCl-0 and isCl-3 samples. Corresponding TA spectra at various delay times are depicted in Fig. 4d, e. For the isCl-0 sample, distinct ground-state bleach (GSB) peaks at 404 nm, 424 nm, and 438 nm signify the co-presence of *n* = 2, *n* = 3, and *n* ≥ 4 phases. In contrast, the isCl-3 sample exhibits GSB peaks at 420 nm and 435 nm, corresponding to *n* = 3 and *n* ≥ 4 phases. Such a blue-shift of each phase agrees well with the fluctuation in the absorption edge of the UV–Vis absorption spectra. The relative presence of each phase was quantified by the amplitude of the transient absorption signal in the initial excitation stage (about 0.6 ps) by Gaussian fitting (Fig. S20c, d, Supporting Information)^48,49^. It can be found that there is an increased proportion of high*-n* domains from 67.11% (isCl-0) to 83.68% (isCl-3). Further analysis of TA kinetics involves extracting time traces at specific wavelengths corresponding to *n* = 2 and *n* ≥ 4 phases for the isCl-0 and isCl-3 sample, as depicted in Fig. 4f, g and Table S4 (Supporting Information). The fast decay time (*τ*_1_) of *n* ≥ 4 phases at 435 nm in the isCl-3 samples (0.88 ps) was notably shorter than that of *n* ≥ 4 phases at 438 nm in the isCl-0 sample (3.37 ps). Such shorter *τ*_1_ within sub-ps indicates a more rapid carrier cooling due to enhanced phase reconstruction in the isCl-3 RDPs, which promotes suppressed nonradiative recombination.

**Fig. 5:**
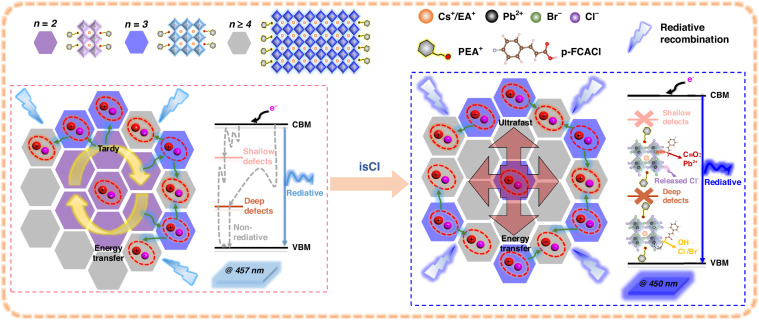
Schematic diagram of the effect of isCl on carrier recombination dynamics and phase reconstruction of RDP film

Based on the above results, it can be concluded that isCl enables the renovation of multiple defects both in the bulk and on the surface of RDPs to enhance radiative recombination based on the results of the PL spectra, PLQYs, TRPL spectra, AS spectra combined with DFT calculation and other chemical analysis. Moreover, phase reconstruction by isCl treatment allows ultrafast energy transfer, as shown in the TA spectra. Increased exciton binding energy also boosts radiative recombination to develop the luminance and prolong the lifetime of the emitters according to the temperature-dependent PL spectra and the TREL spectra. These merits together promote a substantial improvement of charge transfer and recombination processes for enhanced optoelectronic performance of deep-blue devices. Therefore, a picture to understand the impact of isCl on the carrier recombination dynamics and phase reconstruction of RDPs is depicted in Fig. 5. During isCl treatment, released chloride ions renovate halide vacancies both in bulk and on the surface of RDPs, which facilitates enlarged bandgap and blue-shifted emission. Meanwhile, the C=O groups and hydroxy groups in the isCl byproduct, p-FCA, bond with the uncoordinated lead and the halide ions to renovate shallow-state and deep-state defects on the surface. Moreover, the formation of *n* = 2 phases is also thoroughly suppressed by the interaction between the fluorine atoms and organic cations, suggesting ultrafast energy transfer, higher PLQYs, and increased exciton binding energy. Such effective multiple defect renovation and phase reconstruction benefit fast charge transport, enhanced radiative recombination, and superior operation stability to achieve higher luminance and prolonged lifetime of the EL emitters.

## Discussion

In this work, we introduce a novel isCl post-treatment strategy for RDPs. The multifunctional p-FCACl could release chloride ions to fill the halide vacancies both in the bulk and on the surface of RDPs, which contributes to significant blue-shifted emission. Meanwhile, the byproduct of isCl, p-FCA, enables not only renovate shallow-state defects induced by halide vacancies through interactions between the C=O groups and uncoordinated lead but also eliminates deep-state defects induced by halide ion migration through hydroxy-group-derived hydrogen bonds, which simultaneously modifies carrier recommendation dynamics. Moreover, isCl allows the reconstruction of phase distribution by interactions between fluorine atoms and organic cations, thus completely inhibiting *n* = 2 phases, leading to an ultrafast energy transfer and the highest PLQY of 60.9% at 450 nm. Consequently, spectrally stable deep-blue PeLEDs with a record maximum EQE of 6.17% and a four-fold improvement in operation time were demonstrated. This work suggests there is plenty of room for the search for multifunctional molecules for comprehensive construction of high-performance deep-blue perovskite emitters.

## Materials and methods

### Materials

Lead bromide (PbBr_2_), cesium chloride (CsCl), PVK (average M_n_ 25,000–50,000), PVP (average M_w_ 55000), and dimethyl sulfoxide (DMSO) were purchased from Sigma Aldrich. Phenethylamine bromide (PEABr), ethylamine chloride (EACl), and Tri (4-fluorophenyl) phosphine oxide (TFPPO) were bought from Xi’an Yuri Solar Co., Ltd. 2,2’,2”-(1,3,5-Benzinetriyl)-tris(1-phenyl-1-H-benzimidazole) (TPBi) and Liq were purchased from Luminescence Technology. p-Fluorocinnamoyl chloride (p-FCACl), p-Fluorocinnamic acid (p-FCA), and 1, 2-dichloroethane (DCE) were purchased from Aladdin. All the chemicals were utilized directly without any further treatment.

### Preparation of reduced-dimensional perovskite films

CsCl, PbBr_2_, PEABr, and EACl were dissolved according to a molar ratio of 1.1:1:0.8:0.3 in DMSO incorporation with 2 mg mL^−1^ 18-crown-6 for the preparation of 0.15 M precursor solution. Followed by stirring for 4 h in a nitrogen glovebox, the solution was filtered through a polytetrafluoroethylene (PTFE) filter with a pore size of 0.45 μm. The perovskite films were prepared in a nitrogen glovebox via a one-step spin-coating method. In detail, the precursor solution was spin-cast at 5000 rpm for 60 s, and 300 µL solution of p-FCACl and TFPPO (1.5 mg mL^−1^) dissolved in 1,2-dichloroethane used as antisolvent was dropped rapidly on the precursor films after spin coating for 20 s according to the literature. Then, the film was annealed on a hotplate at 70 °C for 3 min.

### Device fabrication

The patterned indium tin oxide (ITO) glass substrate was sequentially sonicated in detergent, deionized water, isopropyl alcohol, and ethanol for 10 min and dried with nitrogen flow. After that, the ITO glass substrate was treated using a UV-zone for 15 min to improve its surface wettability. Then, the substrate was transferred to a nitrogen glovebox to prepare PVK layers (dissolved in chlorobenzene, 6 mg mL^−1^) at 3000 rpm for 30 s, followed by annealing at 100 °C for 15 min. The PVP layers (dissolved in isopropyl alcohol, 2 mg mL^−1^) were spin-coated onto the PVK at 3000 rpm for 30 s, followed by annealing at 100 °C for 5 min. Then, reduced-dimensional perovskite films were prepared by the method described above. Afterward, the TPBi layer (35 nm), Liq layer (2 nm), and Al electrode (120 nm) were deposited on the perovskite films through a thermal evaporator. Finally, the devices with a defined area of 0.04 cm^2^ were fabricated completely.

### Films and device characterization

The scanning electron microscopy (SEM) images were acquired by a field-emission scanning electron microscope (Regulus8100). The atomic force microscope (Bruker) was used to measure atomic force microscopy (AFM) images of perovskite films. The Grazing incidence wide-angle X-ray scattering (GIWAXS) pattern was acquired from Xeuss 3.0 HR WAXS laboratory beamline (XENOCS, France) with X-ray radiation (*λ* = 1.54 Å). The UV–Vis absorption spectrum was measured using a N4S UV–Visible spectrophotometer (Shanghai Instrument & Electronics Analysis Co., Ltd.). The steady-state photoluminescence (PL) spectrum was performed using Horiba Scientific FluoroLog-3 fluorescence spectrophotometer with an excitation wavelength of 365 nm. Time-resolved PL (TRPL) decay measurement was measured using Japan Hamamatsu Quantaurus-Tau C11367 with a 365 nm excition. The PLQYs of the perovskite film were recorded by using a fluorescence spectrometer with an integrated sphere (C9920-02, Hamamatsu Photonics Co., Ltd.). X-ray photoelectron spectroscopy (XPS) measurement was obtained using the Therom Scientific Escalab 250Xi. Ultraviolet photoelectron spectroscopy (UPS) measurement was measured by using the He I (21.22 eV) as an excitation source. Fourier transform infrared (FTIR) measurements were conducted by a Fourier Transform Infrared Spectrometer (Nicolet 6700) in a transmittance mode. Nuclear magnetic resonance (NMR) spectroscopy was collected on a Bruker AVANCE III 600 M using deuterated DMSO as solvent at room temperature. Transient absorption (TA) spectroscopy of the perovskites was performed using the Ultrafast System HELIOS TA spectrometer, where the pump wavelength was 385 nm. Current density-voltage-luminescence (*J–V–L*) curves, EL spectra, EQE, current efficiency (CE), and operational stability measurement of PeLEDs were acquired through the Enlitech measurement system (LQ-100). The thermal admittance spectroscopy (TAS) was conducted by the ZAHNER electrochemical workstation at various temperatures (*T* = 280–310 K) in the dark. The transient EL (TREL) decay curves were measured under an electrical excitation with the pulse (duty cycle of 50%) generated by a generator (Keysight EDU33212A).

### DFT calculations

Density functional theory (DFT) calculations were carried out in the Vienna Ab initio Simulation Package (VASP). The generalized gradient approximation (GGA) and Perdew-Burke-Ernzerhof (PBE) were applied to treat the exchange-correlation function of electrons. The DFT-D3 method was employed to properly consider the long-range van der Waals interaction. A Gamma k-point of (2 × 2 × 1) and cutoff energy of 450 eV were set for all the optimization of perovskite slab models. The geometry optimization was not terminated until all self-consistent field (SCF) calculations were decreased to less than 10^−6 ^eV and the force was smaller than 0.02 eV/Å. The binding energy be between perovskite structure and p-FCA molecule was defined as ${E}_{\left({\rm{binding\; energy}}\right)}$ via following formula: ${E}_{({\rm{binding\; energy}})}={E}_{({\rm{adsorption\; molecule}})}+{E}_{({\rm{perovskite\; structure}})}-{E}_{({\rm{binding\; structure}})}$.

## Supplementary information

Supplementary Information for Multiple Defects Renovation and Phase Reconstruction of Reduced-Dimensional Perovskites via In Situ Chlorination for Efficient Deep-Blue (454 nm) Light-Emitting Diodes

## Data Availability

The data that support the plots within this paper and the other findings of this study are available from the corresponding authors upon reasonable request.
