# Supplementary material for: Multiple defects renovation and phase reconstruction of reduced-dimensional perovskites via in situ chlorination for efficient deep-blue (454 nm) light-emitting diodes
Source: Light Sci Appl. 2025 Feb 26;14:102. doi: 10.1038/s41377-025-01768-3 (PMC11865270; doi:10.1038/s41377-025-01768-3)
Supplement: Supplementary file 1 — Supplementary Information for Multiple Defects Renovation and Phase Reconstruction of Reduced-Dimensional Perovskites via In Situ Chlorination for Efficient Deep-Blue (454 nm) Light-Emitting Diodes [file 41377_2025_1768_MOESM1_ESM.docx]

**Supplementary Information for**

**Multiple Defects Renovation and Phase Reconstruction of Reduced-Dimensional Perovskites via In Situ Chlorination for Efficient Deep-Blue (454 nm) Light-Emitting Diodes**

Mubing Yu^+[1, 2]^, Tingxiao Qin^+[3]^, Gang Gao^*[1, 2]^, Kelei Zu^[4]^, Dongming Zhang^[4]^, Nan Chen^[4]^, Dengke Wang^[4]^, Yong Hua^[5]^, Hong Zhang^[2]^, Yong-Biao Zhao^*[4]^, and Jiaqi Zhu^*[1, 2, 6]^

[1] National Key Laboratory of Science and Technology on Advanced Composites in Special Environments, Harbin Institute of Technology, Harbin 150080, China

[2] Zhengzhou Research Institute, Harbin Institute of Technology, Zhengzhou 450046, China

[3] Beijing Academy of Quantum Information Sciences, Beijing 100193, China

[4] Center for Optoelectronics Engineering Research, School of Physics and Astronomy, Yunnan University, Kunming 650500, China

[5] Yunnan Key Laboratory for Micro/Nano Materials & Technology, School of Materials and Energy, Yunnan University, Kunming 650500, China

[6] Key Laboratory of Micro-systems and Micro-structures Manufacturing Ministry of Education, Harbin Institute of Technology, Harbin 150080, China

[^+^] These authors contributed equally to this work.

*Corresponding authors: Gang Gao (gaogang@hit.edu.cn), Prof. Yong-Biao Zhao (yzhao@ynu.edu.cn), and Prof. Jiaqi Zhu (zhujq@hit.edu.cn)

**Supplementary Note 1.** **The fitting parameters of TREL, TRPL and TA decay curve**

The average life (τ_ave_) of the curves of the reduced-dimensional perovskite films fitted by Equation (1),

$I\left( t \right)=A_{1}exp \left( -\frac{t}{\tau_{1}} \right)+A_{2}exp \left( -\frac{t}{\tau_{2}} \right)$ （1）

where *A_1_* and *A_2_* are the pre-exponential factors, *t* is decay time, *τ_1_* and *τ_2_* are fitted lifetimes. The average lifetime, *τ_ave_*, was calculated using Equation (2),

$\tau_{ave}=\frac{A_{1}{\tau_{1}}^{2}+A_{2}{\tau_{2}}^{2}}{A_{1}\tau_{1}+A_{2}\tau_{2}}$ （2）

The τ_ave_ is determined by the radiative and nonradiative recombination rate constant (*k_r_* and *k_nr_*):

$\tau_{ave}={(k_{r}+k_{nr})}^{-1}$ （3）

Substitute τ_ave_ into the following formula, we can get the radiative recombination rate constant (*k_r_*) and nonradiative recombination rate constants (*k_nr_*):

$PLQY=\frac{k_{r}}{k_{r}+k_{nr}}$ （4）

**Supplementary Note 2.** **Extraction of trap energy level (*E_a_*)**

The trap energy level (*E_a_*) can be derived via the relation by Equation (5) as follows:

$\omega_{0}=\beta T^{2}exp(-\frac{E_{a}}{K_{B}T})$ （5）

where 𝜔_0_ is the characteristic transition (attempt to escape) frequency, which can be defined from the peak value of the [-𝜔×dC/d𝜔] curve. *Β* is temperature independent parameter, *k_B_* is the Boltzmann's constant, and *T* is the temperature, respectively. According to this equation, the Arrhenius plot (ln($\frac{\omega_{0}}{T^{2}}$) =ln$\beta$-$\frac{E_{a}}{K_{B}T}$), and the value of *E_a_* can be obtained from the slope of the Arrhenius plot line.

**Supplementary Note 3.** **Extraction of exciton binding energy (*E_b_*)**

Perovskite materials generally undergo slight structural phase transition with decreasing temperature. However, this aspect does not affect the dielectric confinement problem. The exciton binding energy of the control and target perovskite has been extracted through the fitting procedure. The exciton binding energy was fitted by Equation (6) as follows:

$I\left( T \right)=\frac{I_{0}}{1+Ae^{-\left( \frac{E_{b}}{K_{B}T} \right)}}$ （6）

where *I(T)* is the temperature-dependent integrated PL intensity, *T* is the temperature, *I_0_* is the integrated PL intensity extrapolated at 0 K, *A* is a constant, and *k_B_* is the Boltzmann constant.

**Supplementary Figures**


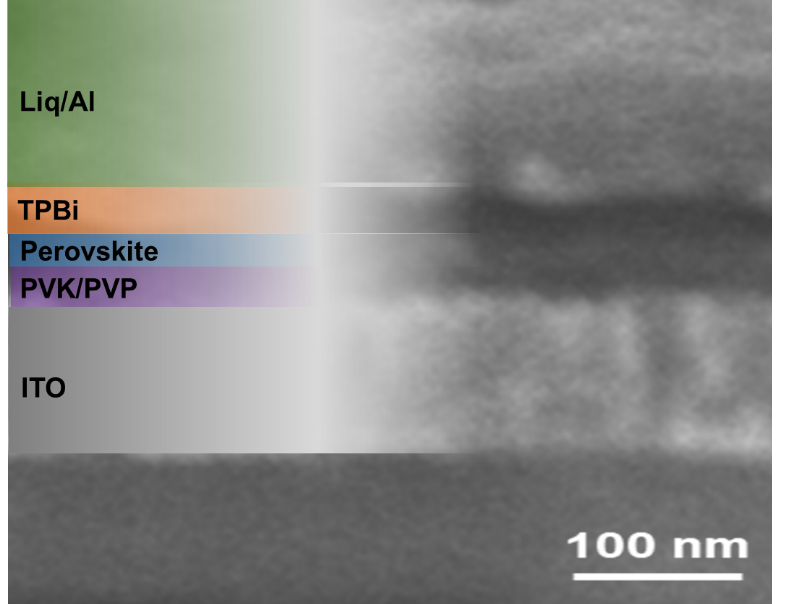


**Figure S1:** Cross-sectional SEM image of the PeLEDs.


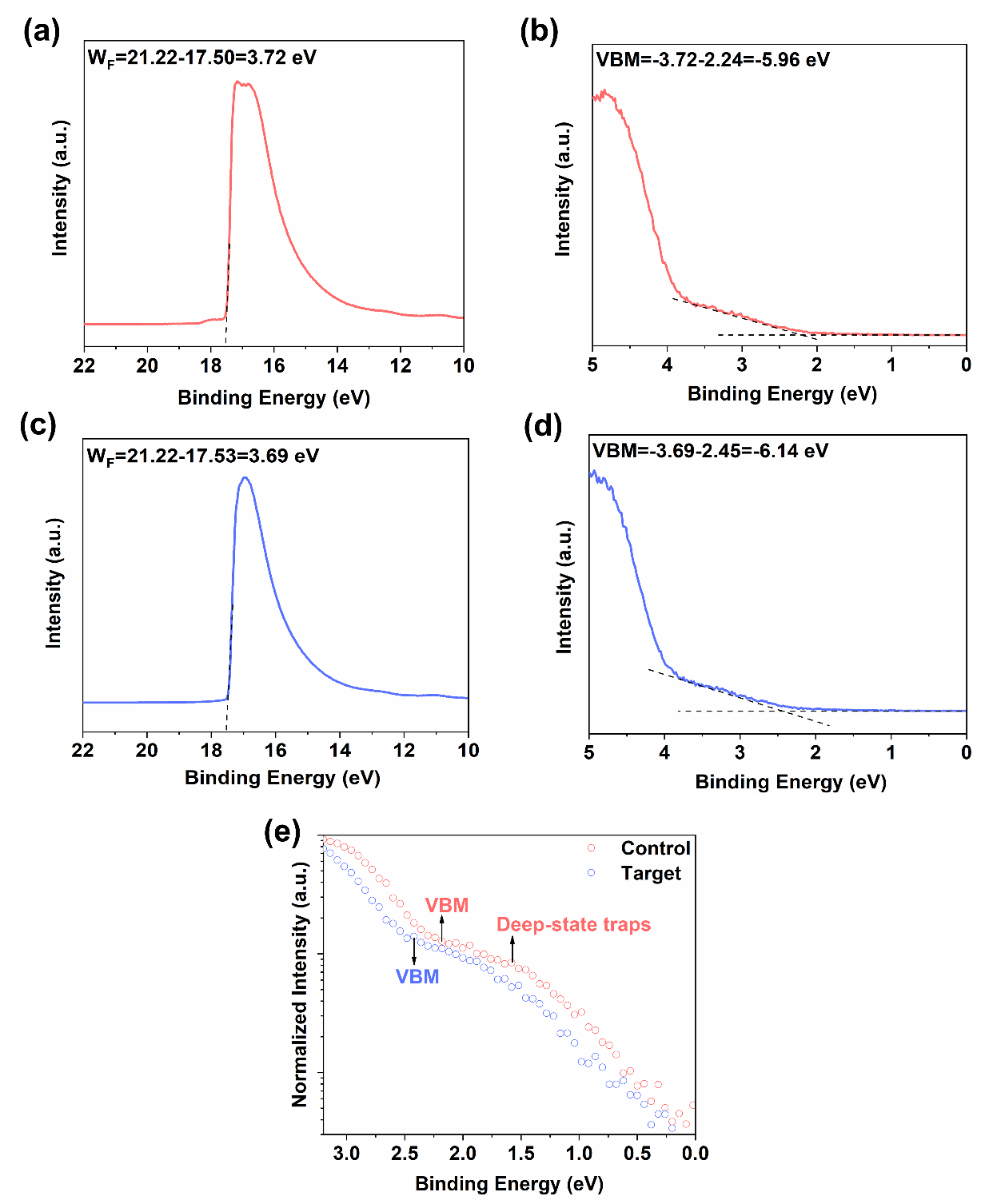


**Figure S2:** UPS spectra of the control RDP film for (a) *E*_F,edge_ and (b) *E*_cut-off_. UPS spectra of the target RDP film for (c) *E*_F,edge_ and (d) *E*_cut-off_. (e) Near Fermi edge region with the semilogarithmic scale in the UPS spectra of the RDP films.


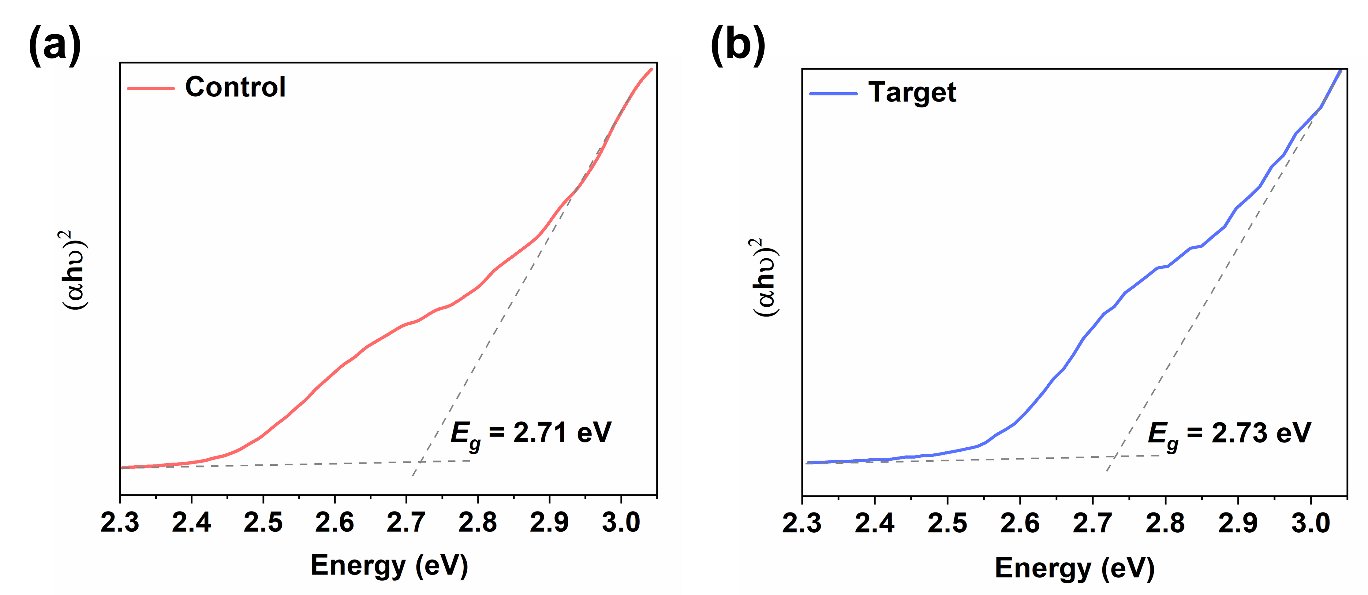


**Figure S3:** Bandgap of the (a) control and (b) target RDP film from steady-state absorption.

**
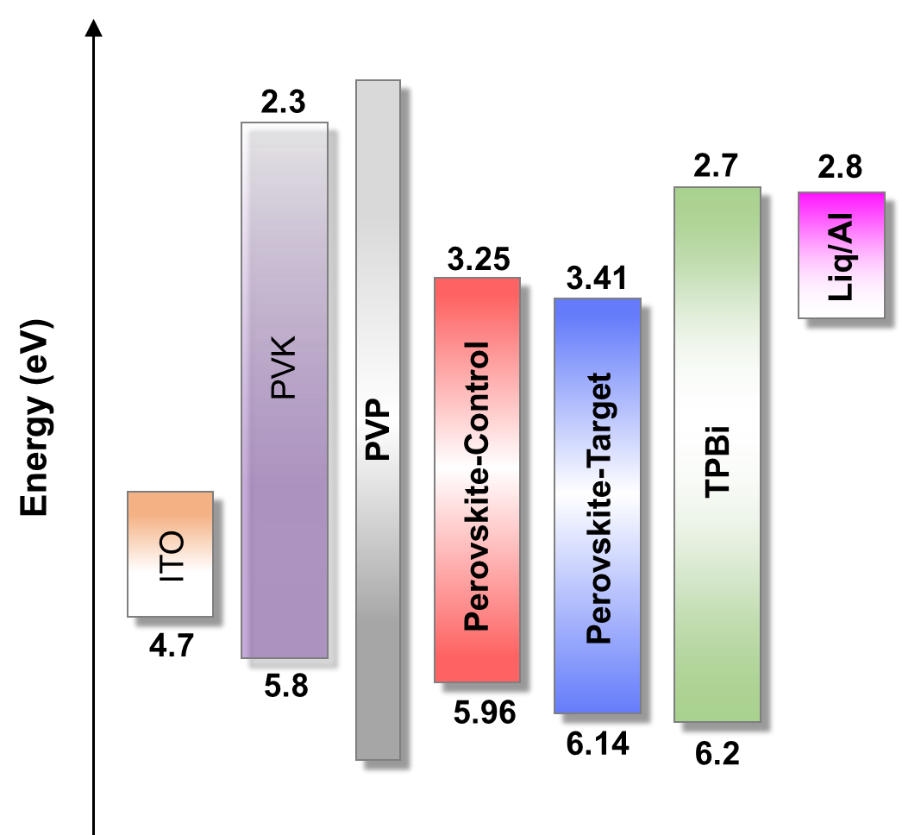
**

**Figure S4:** Energy level alignments of each function layer of the PeLEDs. The energy levels of the RDPs were extracted from Figure S2 as well as Figure S3, and the energy levels of other layers were obtained from the literature.

**

**

**Figure S5:** Comparison of this work with previous deep-blue PeLEDs results with EL emissions ranging from 445 to 458 nm.


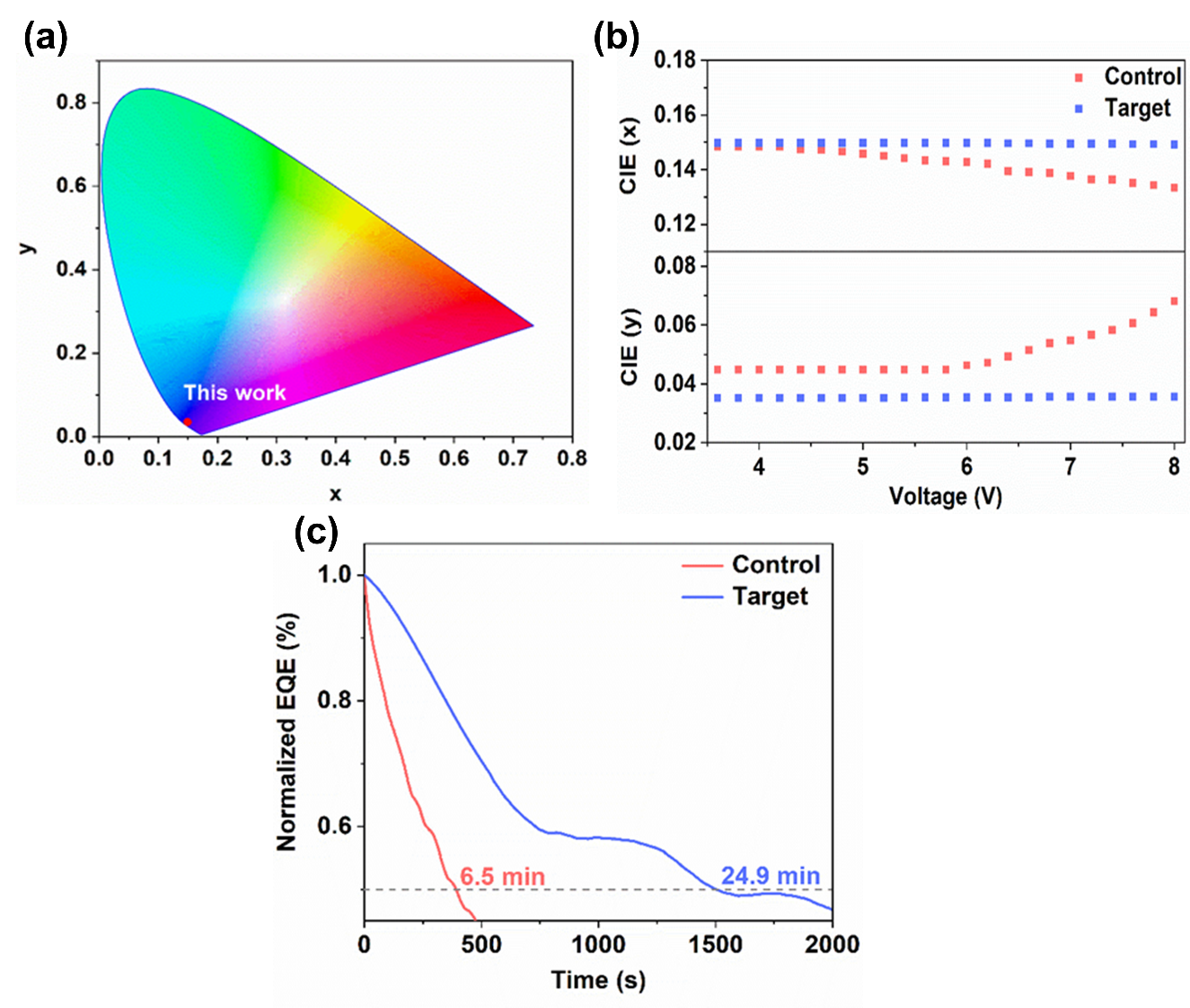


**Figure S6:** (a) CIE coordinate of the target PeLEDs. (b) CIE(x) and CIE(y) versus applied voltages of the PeLEDs. (c) Operation stability of the PeLEDs without encapsulation.





**Figure S7:** ^13^C NMR spectra of p-FCACl and p-FCA dissolved in deuterated DMSO.


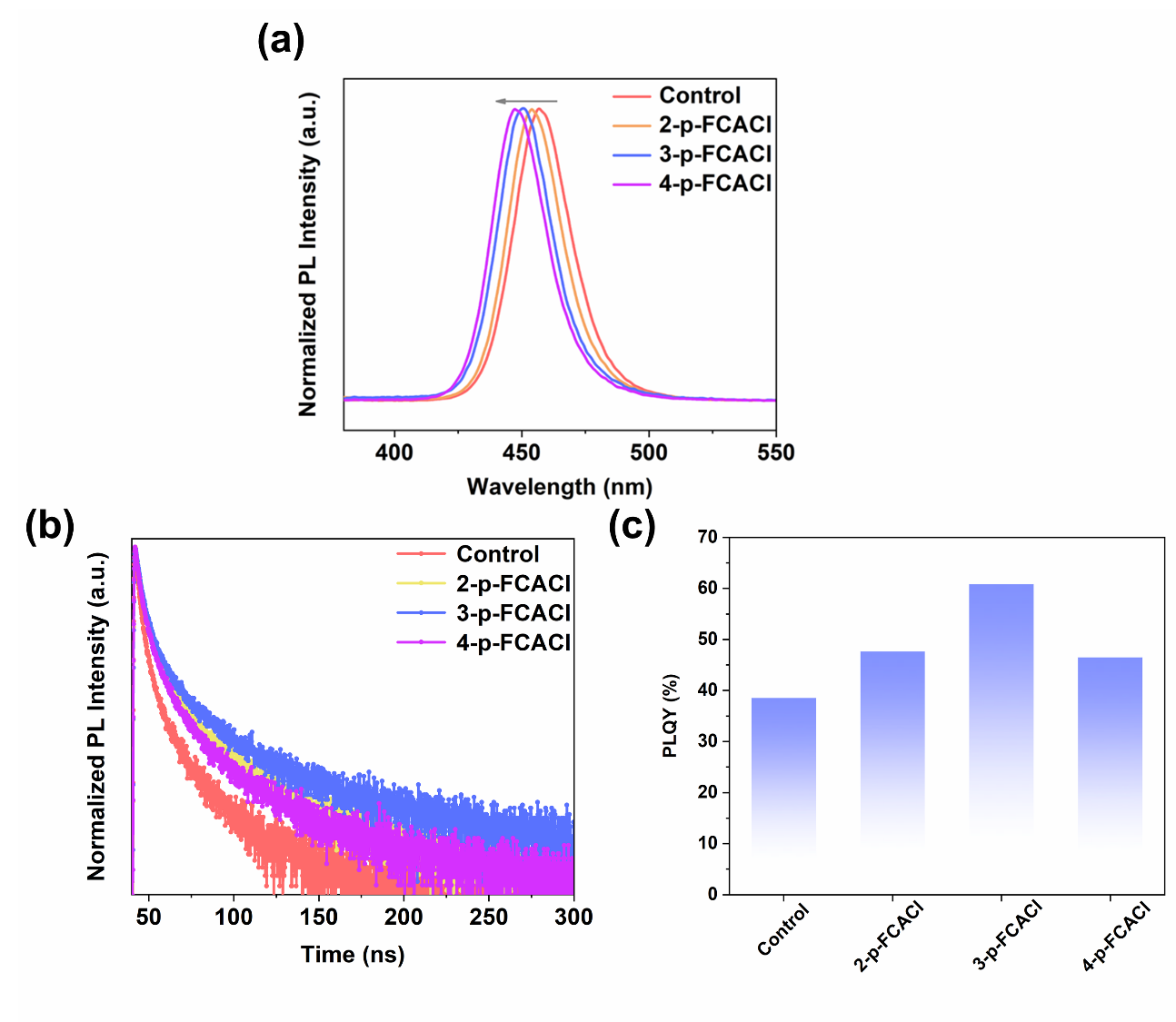


**Figure S8:** PL spectra of the control and p-FCACl-modified RDP film. (b) TRPL spectra and (c) PLQYs of the control and p-FCACl-modified RDP film.


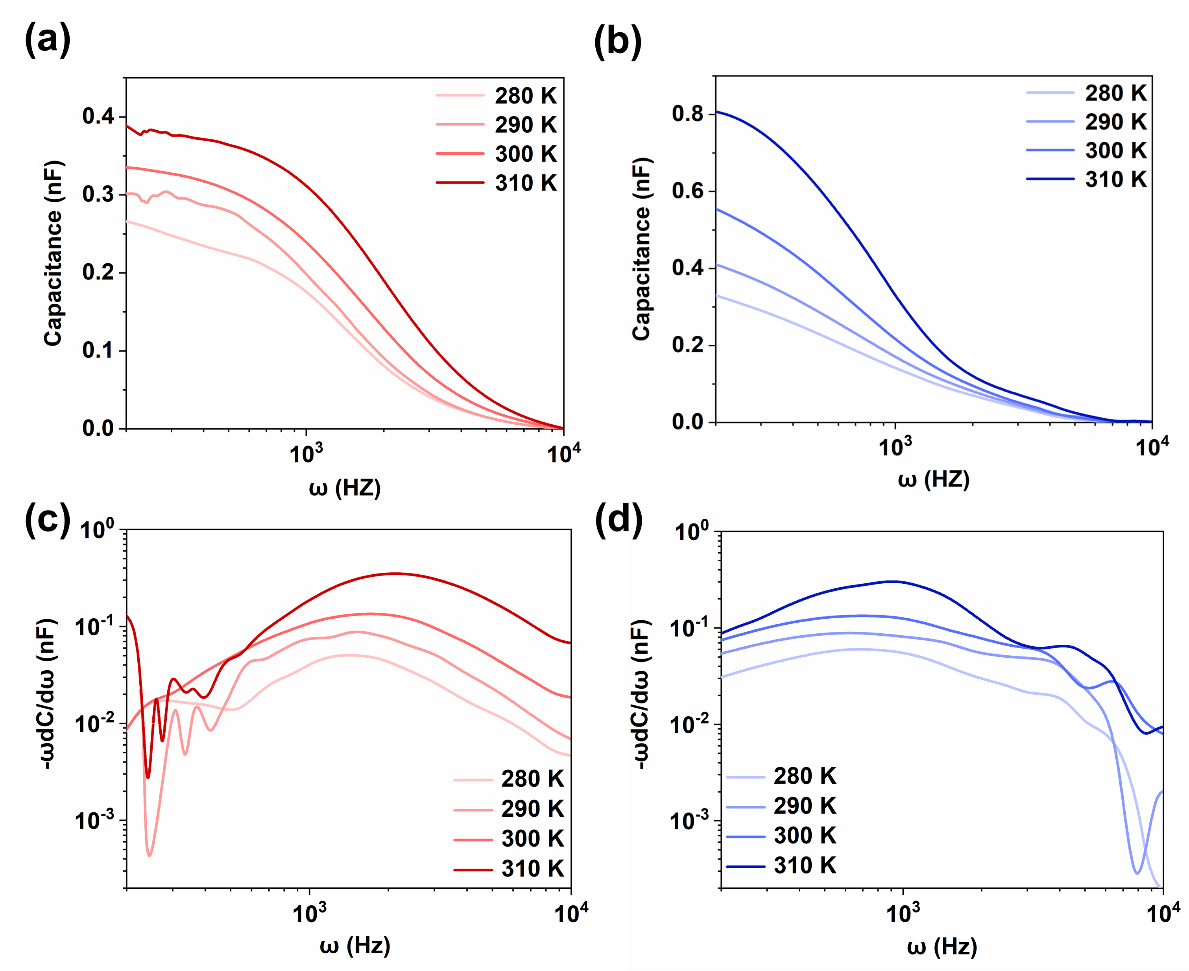


**Figure S9:** Temperature dependent capacitance spectra of the (a) control and (b) target PeLEDs. Derivative specific capacitance with respect to the frequency measured at different temperatures of the (c) control and (d) target PeLEDs. The AC bias is 20 mV.


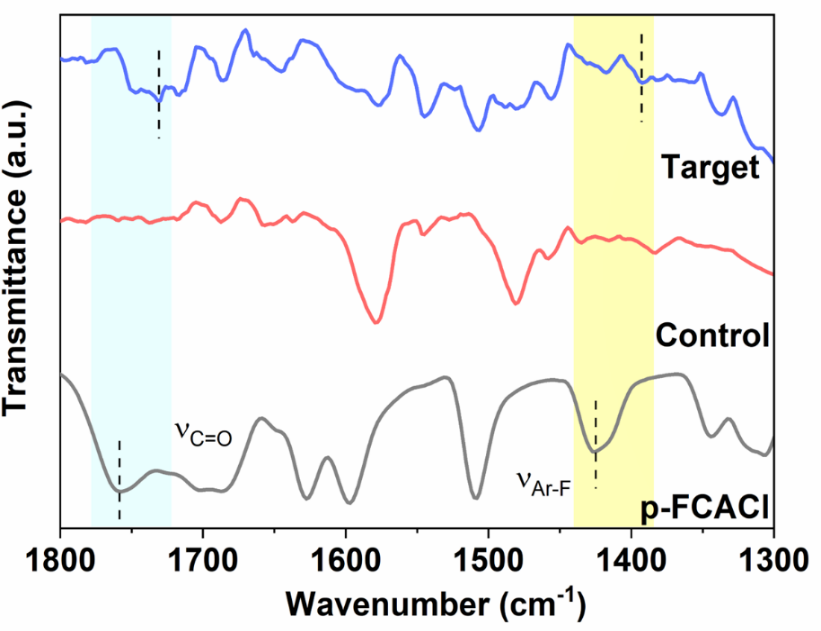


**Figure S10:** FTIR spectra of pure p-FCACl, the control and target RDP film.


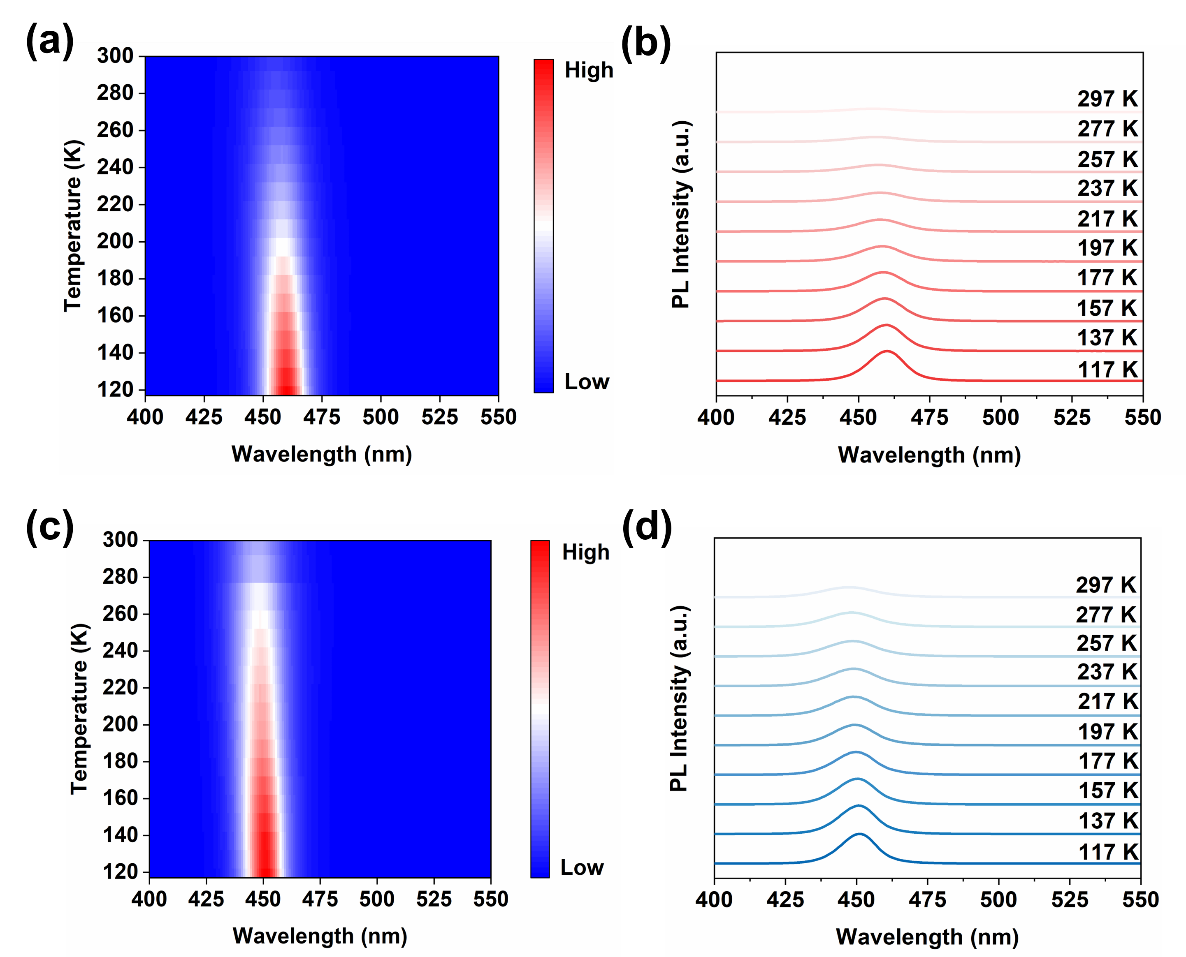


**Figure S11:** (a) Temperature-dependent PL spectra and (b) PL spectra of the control RDP film within the temperature range of 117 K to 297 K. (c) Temperature-dependent PL spectra and (d) PL spectra of the target RDP film within the temperature range of 117 K to 297 K.


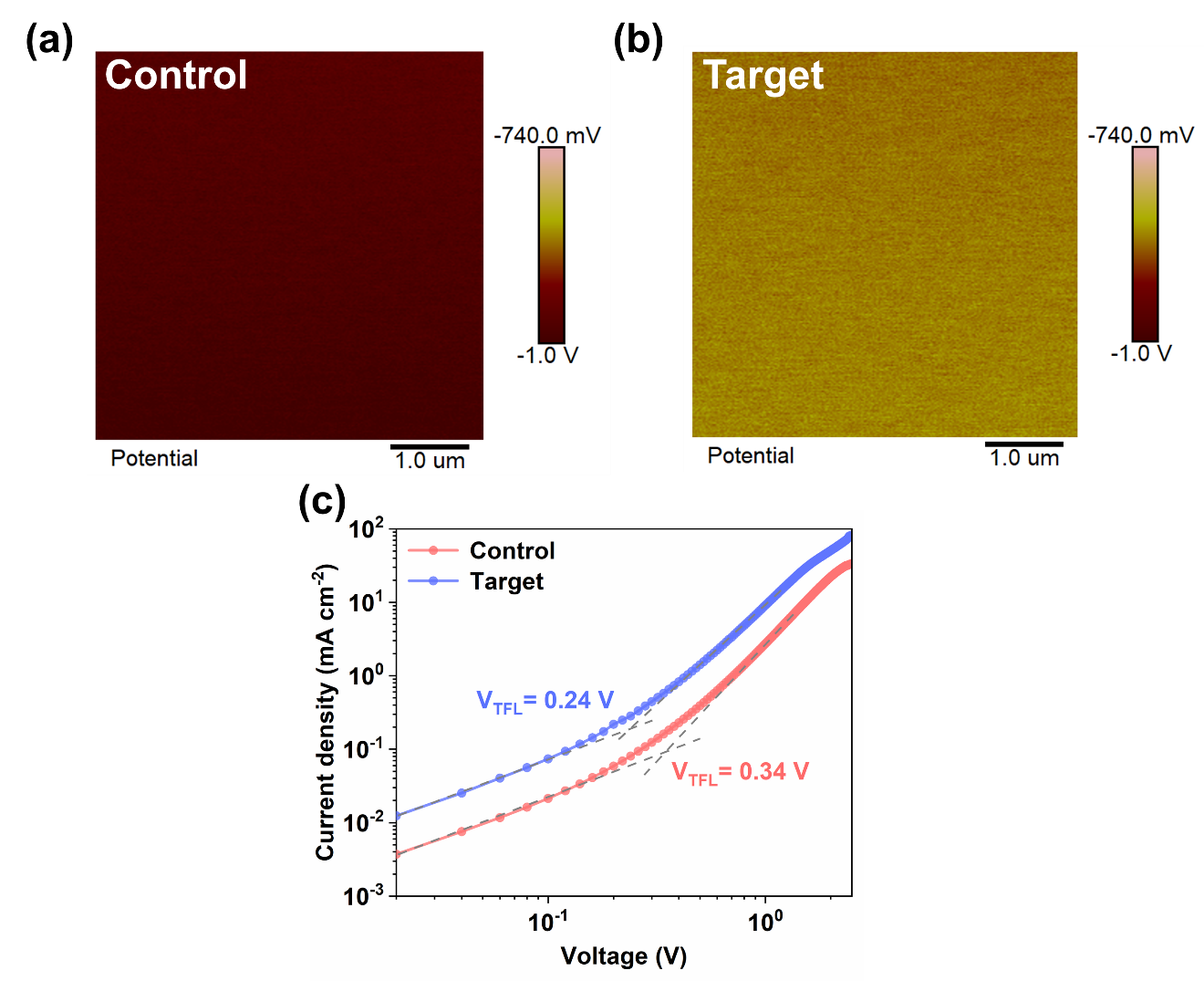


**Figure S12:** KPFM images for the (a) control and (b) target RDP film. (c) The current density-voltage curves of the hole-only devices under dark conditions with the structure of ITO/PVK/PVP/perovskite/CBP/MoO_x_/Al.


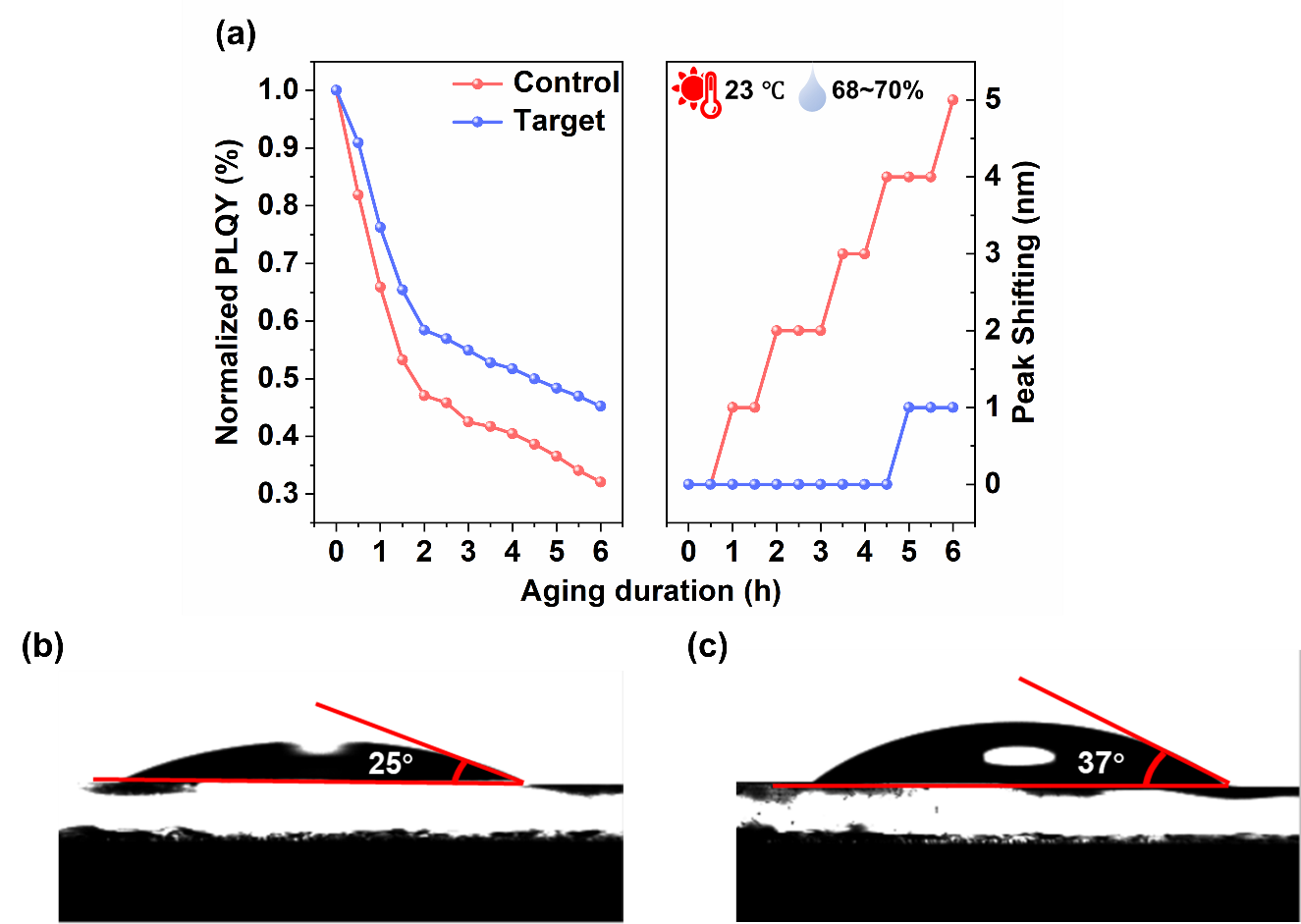


**Figure S13:** (a) Normalized PLQY and emission peak changes with increasing duration of the RDP films. The water contact angle (CA) on the surface of the (b) control and (c) target RDP film.


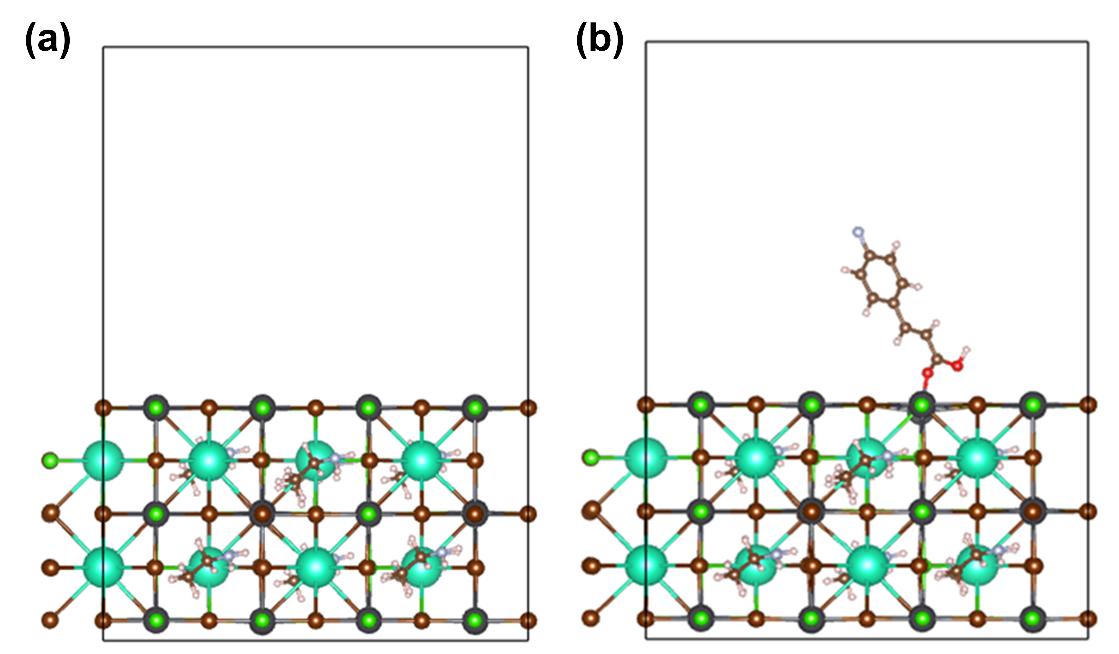


**Figure S14:** Optimized structure of the (a) pristine Cs_0.67_EA_0.33_PbBr_1.2_Cl_1.8_ perovskite and (b) renovated with p-FCA without hydrogen bonds.


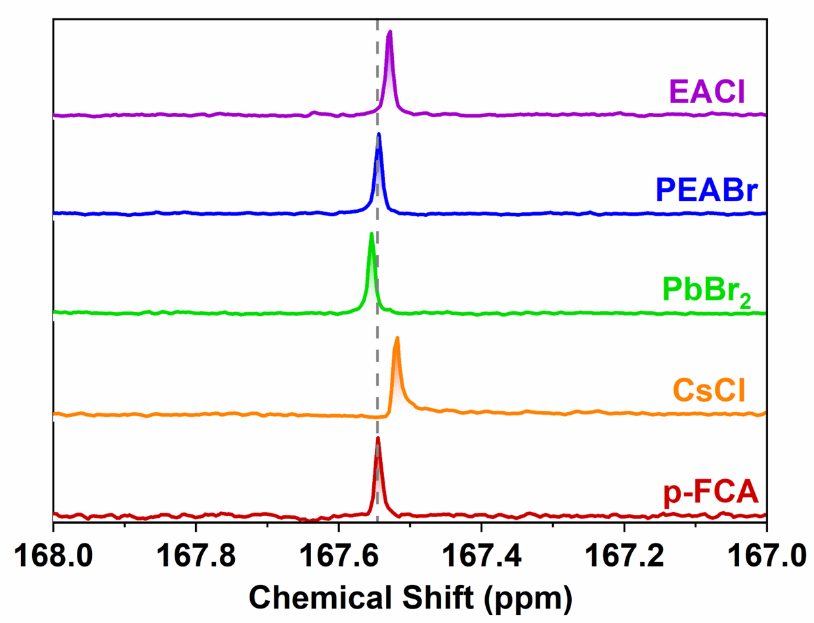


**Figure S15:** ^13^C NMR spectra of pure p-FCA and p-FCA mixed with different perovskite precursor components.


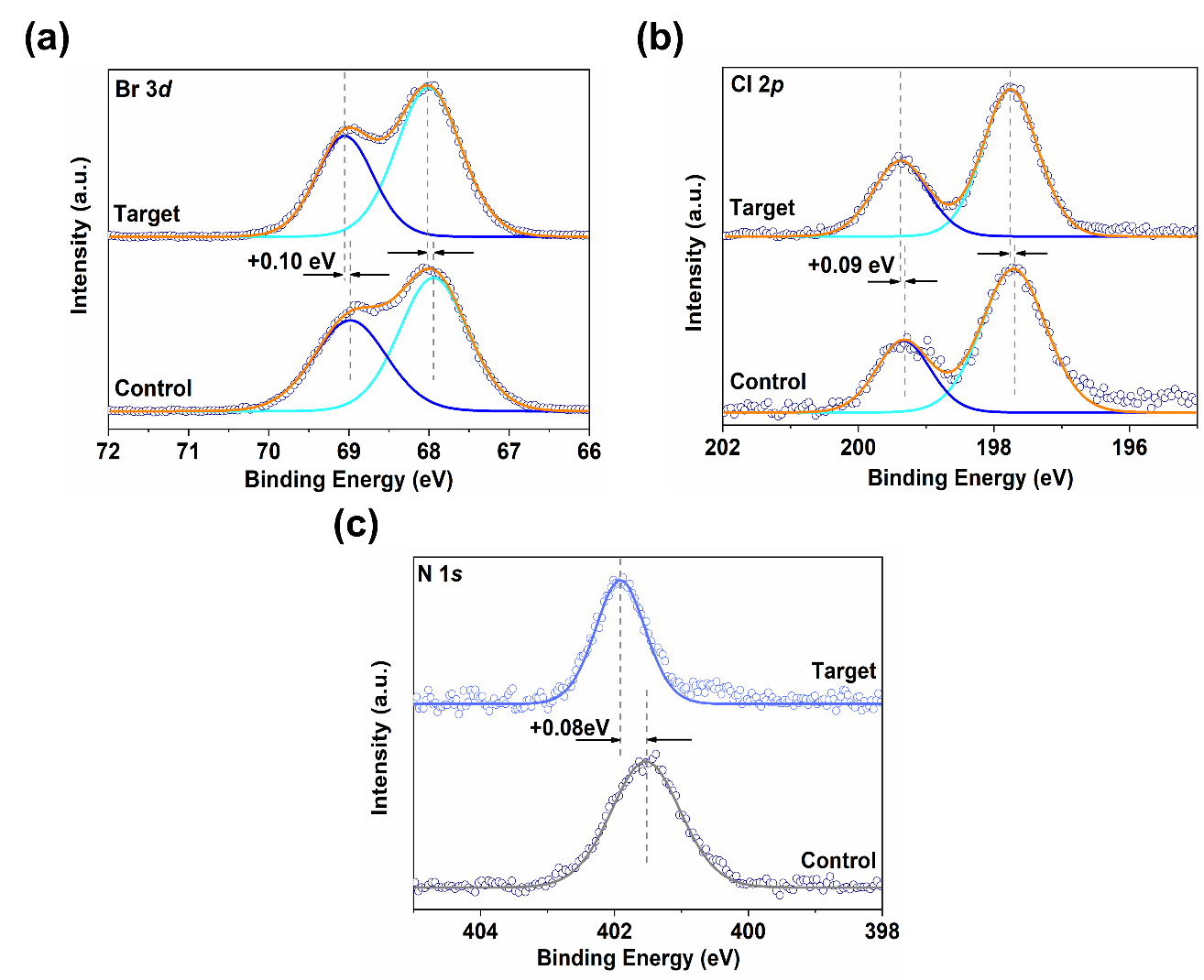


**Figure S16:** Core-level XPS spectra of (a) Br 3*d*, (b) Cl 2*p* and (c) N 1*s* of the RDP films.


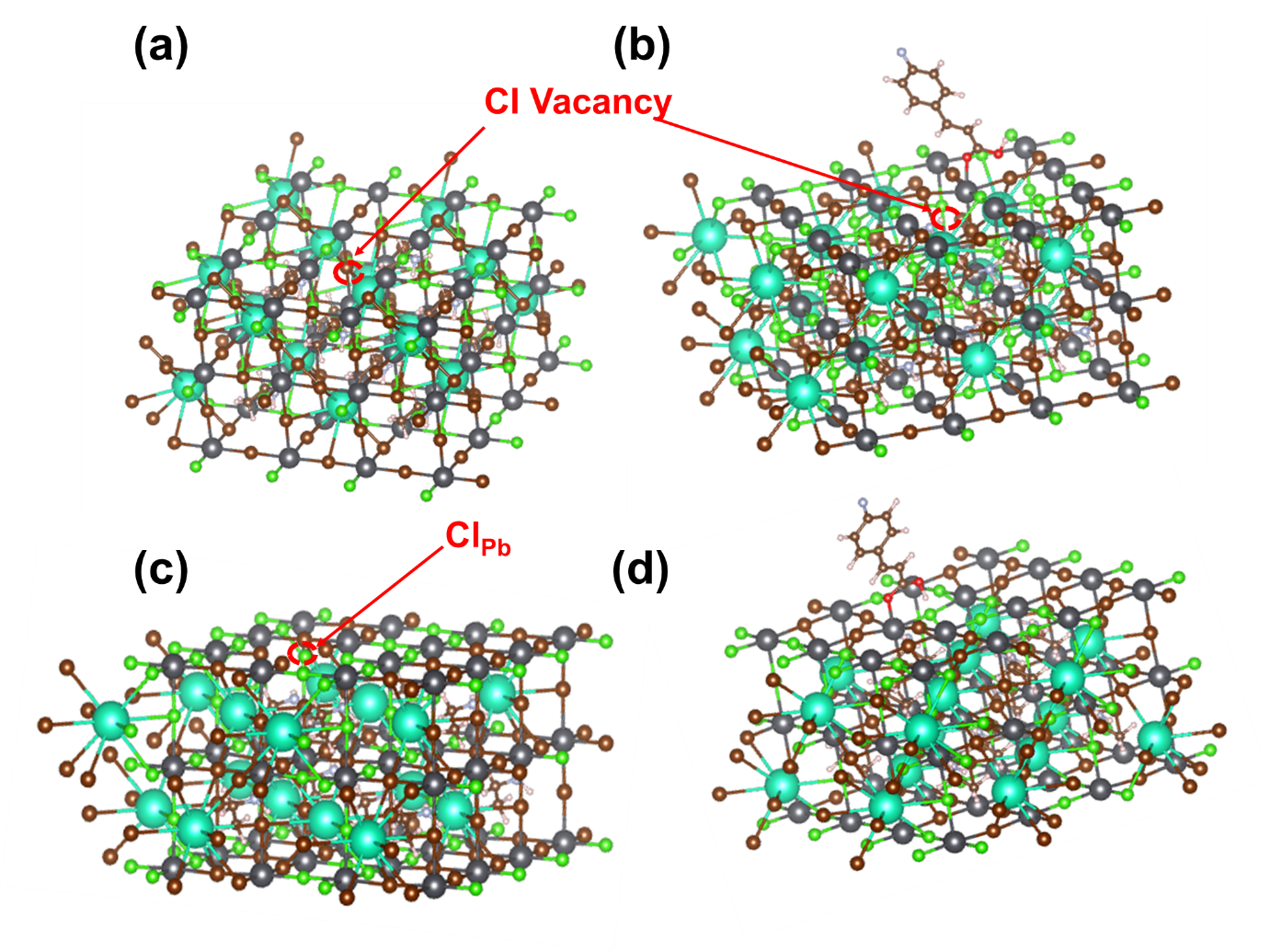


**Figure S17:** Optimized structure of perovskite (a) with a chloride vacancy, (b) renovated by C=O, (c) with a lead-chloride antisite defect, and (d) renovated by -OH.


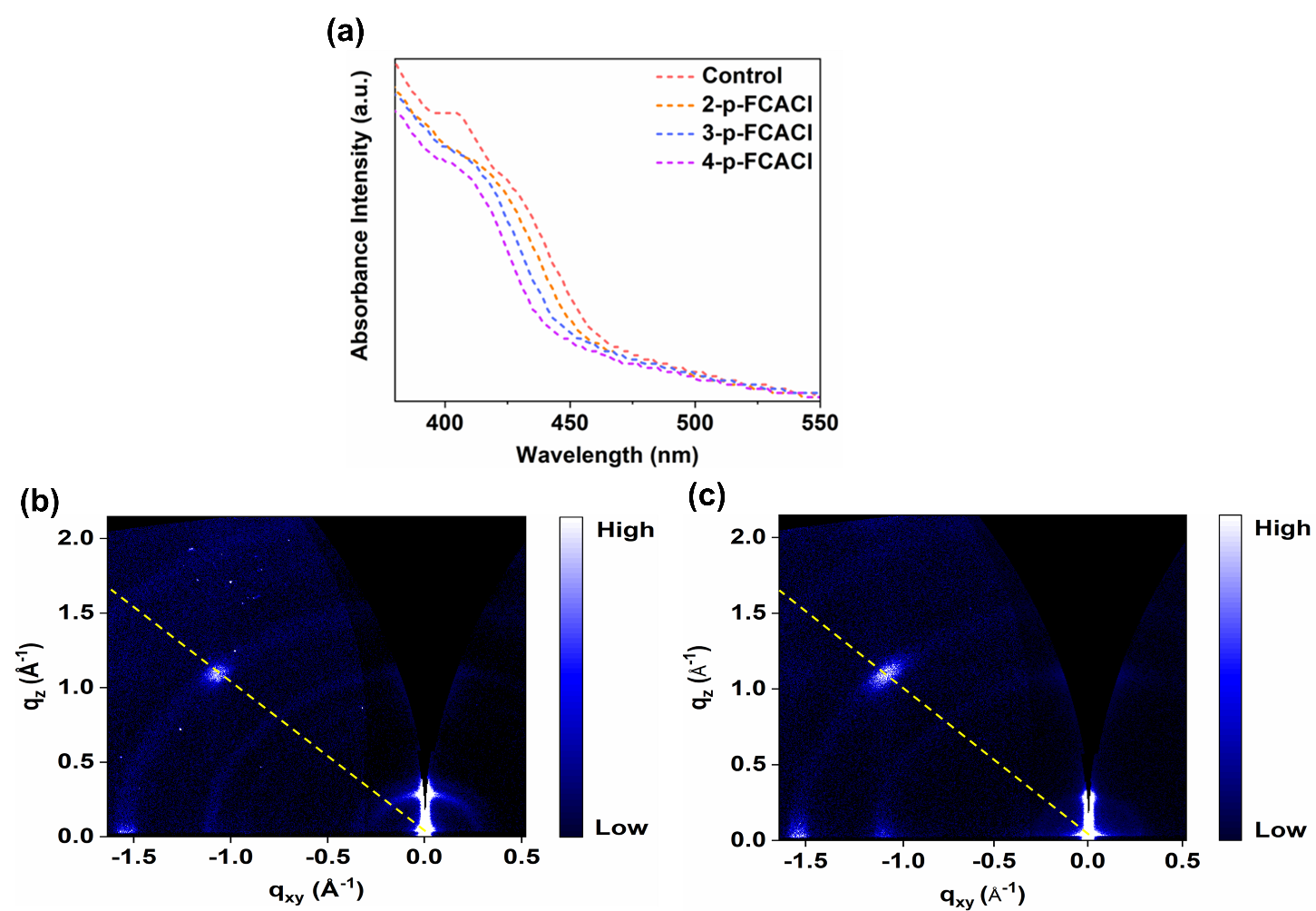


**Figure S18:** (a) Light absorption spectra of the RDP films. GIWAXS of the (b) control and (c) target RDP film.


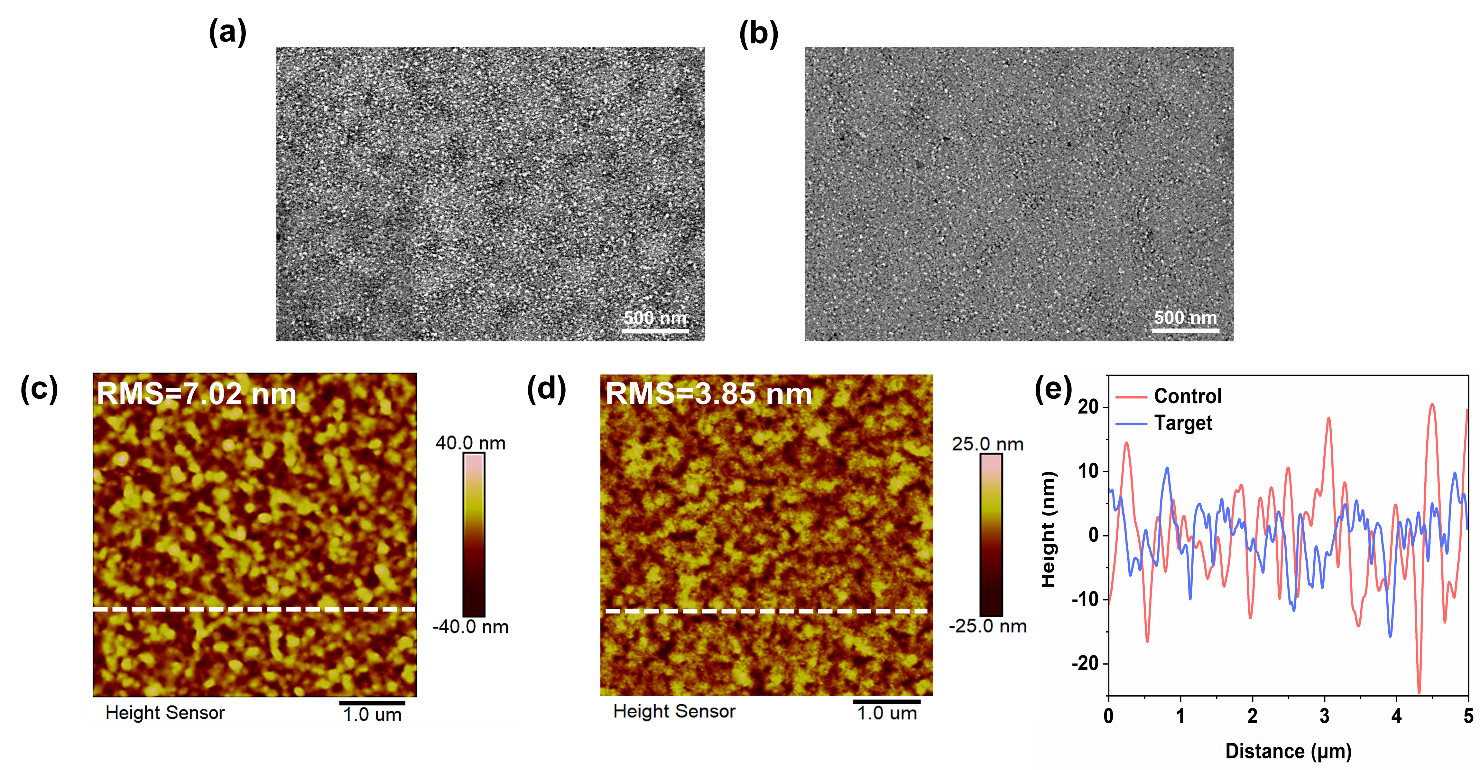


**Figure S19:** The SEM images of the (a) control and (b) target RDP film. The AFM images of the (c) control and (d) target RDP film. (e) Line profile images of AFM images of the RDP films along the line shown in (c) and (d).


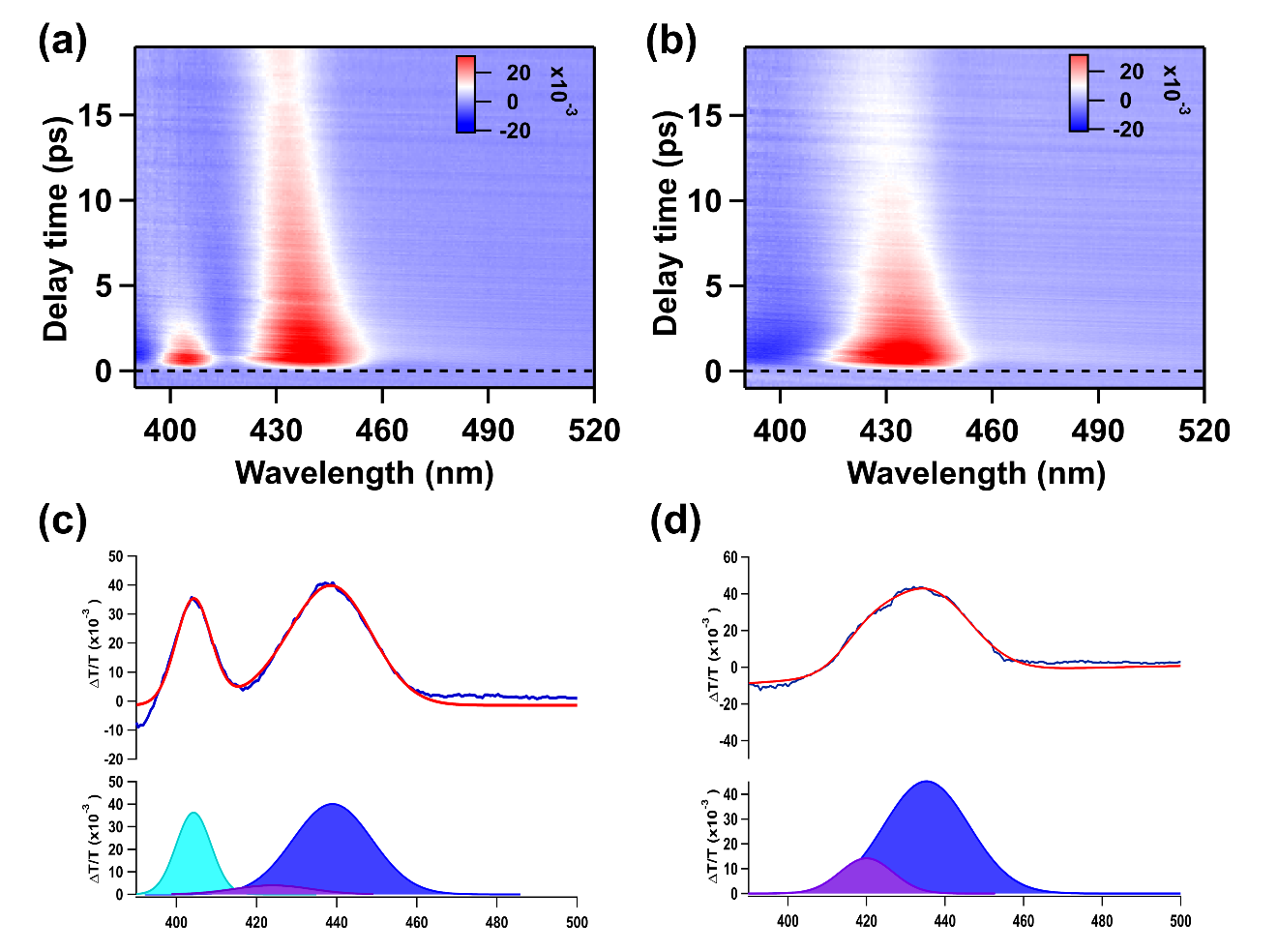


**Figure S20:** TA color maps of the (a) control and (b) target RDP film. Gaussian fitting of the transient absorption signal in the initial excitation stage (about 0.6 ps) of the (c) control and (d) target RDP film.

**Supplementary Tables**

**Table S1:** TREL lifetime characteristics of the PeLEDs.

| x | τ_1_  [μs] | A_1_ | τ_2_  [μs] | A_2_ | τ_avg_  [μs] |
| --- | --- | --- | --- | --- | --- |
| Control | 0.32 | 2.25 | 2.88 | 0.15 | 1.28 |
| Target | 0.52 | 1.72 | 6.17 | 0.11 | 2.96 |

**Table S2:** isCl reaction energy of p-FCACl calculated by DFT.

| isCl | Reaction Energy (eV) |
| --- | --- |
| p-FCACl + H_2_O → p-FCA + Cl^-^ + H^+^ | -0.24 |

Notes: p-FCACl and p-FCA represent p-Fluorocinnamoyl and p-Fluorocinnamic acid, respectively.

**Table S3:** TRPL lifetime characteristics of x-p-FCACl-modified RDP films.

| x | τ_1_  [ns] | A_1_ | τ_2_  [ns] | A_2_ | τ_avg_  [ns] |
| --- | --- | --- | --- | --- | --- |
| Control | 1.92 | 1.54 | 9.08 | 0.19 | 4.55 |
| 2-p-FCACl | 3.08 | 1.12 | 15.74 | 0.20 | 9.12 |
| Target | 3.23 | 1.19 | 19.42 | 0.18 | 10.94 |
| 4-p-FCACl | 2.80 | 1.12 | 14.06 | 0.20 | 8.12 |

**Table S4.** The kinetics fitting parameters of each GSB of the RDP films.

| x |  | τ_1_  [ps] | A_1_ | τ_2_  [ps] | A_2_ |
| --- | --- | --- | --- | --- | --- |
| Control | 404 nm | 1.04 | 0.71 | 3.17 | 0.35 |
|  | 438 nm | 3.37 | 0.40 | 12.84 | 0.55 |
| Target | 435 nm | 0.88 | 0.38 | 5.25 | 0.52 |

**References**

1. Hong, Y. J. et al. Perovskite Nanocrystals Protected by Hermetically Sealing for Highly Bright and Stable Deep-Blue Light-Emitting Diodes. *Advanced Science* **10**, 2302906 (2023).

2. Gao, L. et al. Eliminating Nanocrystal Surface Light Loss and Ion Migration to Achieve Bright Mixed-Halide Blue Perovskite LEDs. *ACS Applied Materials & Interfaces* **15**, 18125-18133 (2023).

3. Baek, S. et al. Highly Stable All‐Inorganic Perovskite Quantum Dots Using a ZnX_2_‐Trioctylphosphine‐Oxide: Application for High‐Performance Full‐Color Light‐Emitting Diode. *Advanced Optical Materials* **8**, 1901897 (2020).

4. Wang, L. T. et al. Colloidal Synthesis of Ternary Copper Halide Nanocrystals for High-Efficiency Deep-Blue Light-Emitting Diodes with a Half-Lifetime above 100 h. *Nano Letters* **20**, 3568-3576 (2020).

5. Qin, Z. P. et al. Co-regulation strategy dominated by double short molecules permitting the regrowth of quantum dots for efficient deep-blue perovskite light-emitting diodes. *Nano Energy* **121**, 109263 (2024).

6. Xia, Y. et al. Vertically Concentrated Quantum Wells Enabling Highly Efficient Deep-Blue Perovskite Light-Emitting Diodes. *Angewandte Chemie International Edition* **63**, e202403739 (2024).

7. Zou, G. R. et al. Color‐Stable Deep‐Blue Perovskite Light‐Emitting Diodes Based on Organotrichlorosilane Post‐Treatment. *Advanced Functional Materials* **31**, 2103219 (2021).

8. Zhou, Y. H. et al. Stabilized Low-Dimensional Species for Deep-Blue Perovskite Light-Emitting Diodes with EQE Approaching 3.4%. *Journal of the American Chemical Society* **144**, 18470-18478 (2022).

9. Dong, J. C. et al. Deep-Blue Electroluminescence of Perovskites with Reduced Dimensionality Achieved by Manipulating Adsorption-Energy Differences. *Angewandte Chemie International Edition* **61**, e202210322 (2022).

10. Yuan, S. et al. Efficient and Spectrally Stable Blue Perovskite Light-Emitting Diodes Employing a Cationic π-Conjugated Polymer. *Advanced Materials* **33**, 2103640 (2021).
